# Characterization of C2H2 superfamily expansions in cephalopods and their contribution to nervous system evolution

**DOI:** 10.1016/j.isci.2025.113561

**Published:** 2025-09-12

**Authors:** Christina Holzinger, Elena A. Ritschard, Pamela Imperadore, Giovanna Ponte, Caroline B. Albertin, Graziano Fiorito, Oleg Simakov

**Affiliations:** 1 Department of Neuroscience and Developmental Biology, University of Vienna, Vienna, Austria; 2 Department of Biology and Evolution of Marine Organisms, Stazione Zoologica Anton Dohrn, Napoli, Italy; 3 Eugene Bell Center for Regenerative Biology and Tissue Engineering, Marine Biological Laboratory, Woods Hole, MA 02543, USA

**Keywords:** Zoology, Neuroscience, Evolutionary biology, Phylogeny

## Abstract

Zinc finger proteins comprise a large family of transcription factors involved in diverse processes, from suppression of transposable elements to regulation of neuronal development. In some clades, including vertebrates and coleoid cephalopods, members of the Cys2His2-type (C2H2) class of zinc fingers were shown to be expanded and likely relevant for neuron differentiation. Using genomic data from three octopus and three squid species, we show that C2H2 evolutionary history can be subdivided into duplication steps: (1) the coleoid expansions, (2) octopus-specific expansions, and (3) squid-specific expansions. While the overall expression patterns are consistent among those expansions and are associated with nervous tissues, the coleoid-specific duplicates showed the highest expression levels compared to all other C2H2 genes. We also found that C2H2 duplicates segregated into different co-expression modules, some associated with other duplicated gene families. We propose a scenario of functional divergence of C2H2 duplicates related to regulation within the cephalopod nervous system.

## Highlights

• C2H2 zinc finger complement is characterized across coleoid cephalopod species
• Coleoid, squid, and octopus expansions of this superfamily can be identified
• Coleoid-specific C2H2 expansion has strongest expression levels
• Expression module analysis reveals functional divergence of C2H2 paralogs

## Graphical abstract

**Figure:**
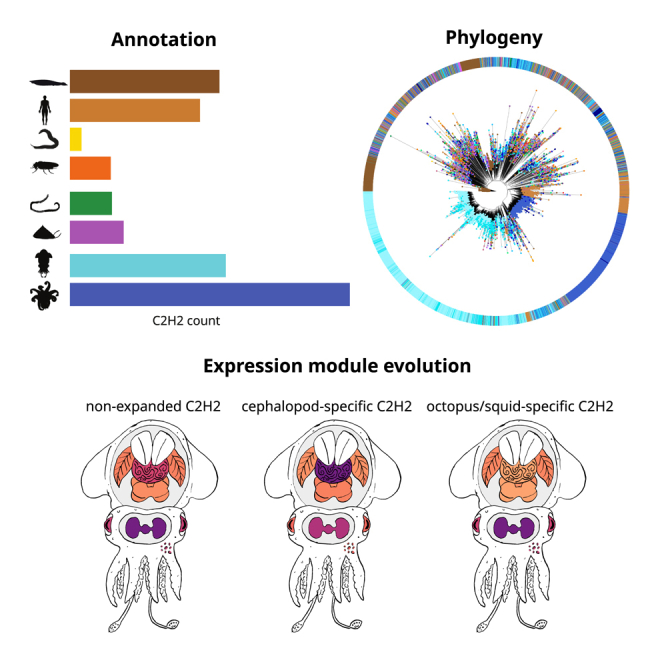

## Introduction

Cephalopods are a class of active predatory molluscs, exhibiting a distinct suite of morphological and behavioral characteristics that clearly set them apart from other taxa in this phylum. Among the distinguishing novelties, we consider the development of flexible arms with suckers - which provide tactile and chemosensory information from the environment^1^ - and the ability to camouflage through the contraction and expansion of chromatophore cells,^2^ both of which may have contributed to the evolution of cephalopods’ agile predatory skills.^1^^,^^3^ These behaviors are driven by a large and elaborate nervous system, the largest among invertebrates, which has evolved to acquire great complexity, even paralleling vertebrates.^4^ Because of their elaborated behavioral repertoire and morphological novelties, cephalopods are ideal candidate organisms for investigating the interconnectedness of evolutionary mechanisms in forming innovations and their underlying genomic contribution,^5^ especially with a focus on nervous system evolution.

One of the major mechanisms of genome evolution is gene and genome duplication, which, through creation of redundancy, can enable organisms to adapt to surrounding influences and develop complex regulatory gene networks.^5^ The redundancy in newly duplicated genes allows for divergence, without the risk of losing the original gene function, and the fate of the copies mostly depends on selection pressure. Two of the hypotheses why redundant genes are kept in the genome are neo- and subfunctionalization.^5^^,^^6^ Neofunctionalization postulates that one copy retains the original function, while the other one accumulates substitutions and gains a completely new function. In contrast, subfunctionalization posits that redundant duplicated genes evolve to maintain together the original function by dividing the work. Previous genome studies^7^^,^^8^ have found that cephalopods show several gene family expansions through duplication, which are thought to play a crucial role in the development and maintenance of the nervous system. Some of these expansions were previously believed to be only present in vertebrates, including the Cys2His2-type (C2H2) zinc finger transcription factors, protocadherins and G-protein coupled receptors (GPCRs). While protocadherins and GPCR expansions have been already thoroughly studied *in silico*,^9^^,^^10^ the evolutionary history and underlying mechanisms of the cephalopod C2H2 repertoire and its functional relation to other gene family expansions remain elusive.

C2H2 zinc finger proteins are the largest family of DNA and chromatin binding transcription factors in many metazoans,^11^ including humans, and are usually composed of groups of tandem zinc finger domains.^12^ Combinations of these domains and their interactions allow for a large number of potential targets, impacting a wide range of cellular processes.^13^ In humans, C2H2 transcription factors are prominently involved in early embryonic development and cell fate determination, especially during neurogenesis.^14^ Due to the large number of these genes, further functional dissection is often problematic.^15^ Nevertheless, expansions of subfamilies of C2H2, such as the KRAB subfamily, have been associated with various developmental novelties, such as placental evolution and live-birth in vertebrates.^15^^,^^16^ Their outstanding diversification in many metazoan genomes may thus hint at an adaptive role of these transcription factors driving evolutionary innovations.^13^

In this study, the evolution of the C2H2 gene repertoire in coleoid cephalopods was assessed by constructing a phylogenetic tree of the gene family using a broad sampling of 20 species, including seven cephalopods. This approach allowed us to differentiate between squid-, octopus-, and cephalopod-specific (taxon specific) expansions. Additionally, tissue expression was compared between C2H2-expanded and non-expanded genes in four cephalopod species with transcriptomic data available to assess the functional changes of these duplicated genes. To further shed light into the putative regulatory landscape of C2H2s, the genes were assigned to co-expression modules. Lastly, correlated C2H2 expansions with other large gene families in cephalopods were explored by using Gene Ontology (GO) term enrichment analysis of co-expression modules. With this combined approach, it was found that expanded C2H2 genes maintained their expression in the brain in cephalopods, similar to the more conserved non-expanded genes. However, expanded C2H2s contributed to different expression modules, suggesting an underlying shift of gene regulation during the expansion of this gene family. These results contrast with the other hallmark cephalopod expansion of GPCRs, where the expression of expanded genes shifted to tissues outside of the brain, which could hint at a functional correlation between expanded gene families during development of the cephalopod nervous system. This work contributes to a deeper insight into C2H2 expansion in cephalopods and builds a foundation for future forays into genomic innovation and its relationship to morphological novelties.

## Results

### Phylogenetic analysis reveals C2H2 expansions at different time points during cephalopod evolution

**Figure 1:**
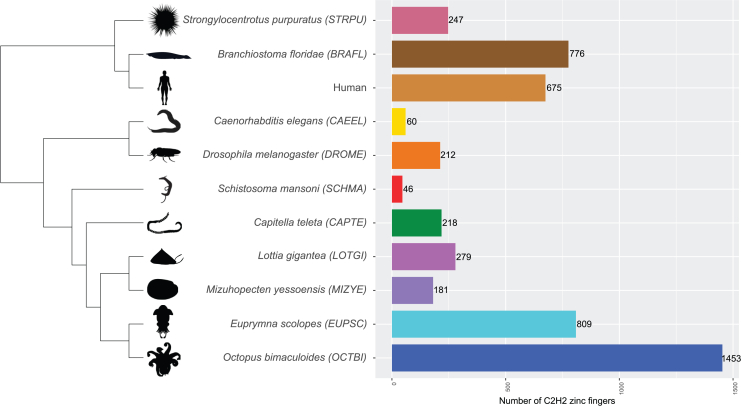
Number of C2H2 protein sequences per species An assortment of species representing big phyla across the bilaterian clade was chosen from the 20 species used for tree construction to highlight C2H2 expansion in cephalopods. Species were roughly sorted according to their genetic distance, with the three uppermost species (HUMAN, BRAFL, and STRPU) being Deuterostomia and therefore acting as sister clade to the other Protostome species, including the coleoid cephalopods. The complete species sampling and their corresponding number of C2H2 protein sequences can be found in Figure S1.

The final filtered and trimmed dataset (available from the Dryad Digital Repository, https://doi.org/10.5061/dryad.jh9w0vtfb) used for tree construction consisted of 8,888 C2H2 zinc-finger protein sequences, 5,603 of which belonged to cephalopod species (Figures 1 and S1). The majority of C2H2 protein sequences corresponded to *Doryteuthis pealeii* (2,442 sequences), followed by *Octopus bimaculoides* (1,453 sequences) and *Euprymna scolopes* (809 sequences). In previous studies,^7^^,^^8^^,^^17^ coleoid cephalopods showed an increased number of C2H2 zinc fingers compared to other protostome species as well as deuterostome species, some of which have also exhibited zinc finger expansions (e.g., human^18^ and *Branchiostoma floridae*). Following our results, for example, C2H2s comprise 4% of the genes in the genome (1,400 to around 33,000 total number of genes) in *O. bimaculoides*, which is comparable to the 3% in humans.^19^ In contrast, *C. elegans* displays 0.9% of C2H2s in its genome.^19^

**Figure 2:**
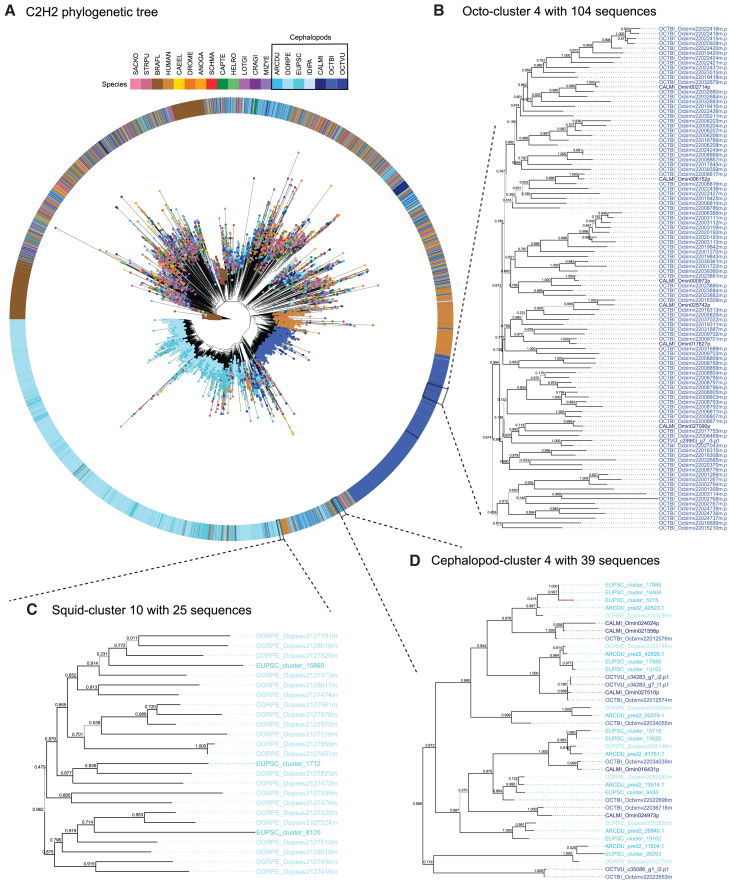
Phylogenetic tree of the C2H2 zinc finger gene family constructed with 20 species (A) Complete tree containing 8,888 C2H2 zinc-finger protein sequences. Abbreviations are as follows: ANOGA, *Anopheles gambiae*; ARCDU, *Architeuthis dux*; BRAFL, *Branchiostoma floridae*; CAEEL, *Caenorhabditis elegans*; CALMI, *Callistoctopus minor*; CAPTE, *Capitella teleta*; CRAGI, *Crassostrea gigas*; DORPE, *Doryteuthis pealeii*; DROME, *Drosophila melanogaster*; EUPSC, *Euprymna scolopes*; HELRO, *Helobdella robusta*; HUMAN, IDIPA, *Idiosepius paradoxus*; LOTGI, *Lottia gigantea*; MIZYE, *Mizuhopecten yessoensis*; OCTBI, *Octopus bimaculoides*; OCTVU, *Octopus vulgaris*; SACKO, *Saccoglossus kowalevskii*; SCHMA, *Schistosoma mansoni*; and STRPU, *Strongylocentrotus purpuratus.* Examples of expansion clusters are shown as zoom-ins in (B), (C), and (D). Node support values are written above the branches in the trees. All expansion clusters can be additionally found in the Dryad Repository (https://doi.org/10.5061/dryad.jh9w0vtfb). (B) Octopus-specific expansion cluster containing 104 sequences. (C) Squid-specific expansion cluster containing 25 sequences. (D) Cephalopod-specific expansion cluster containing 39 sequences.

To investigate the C2H2 zinc finger gene expansion in cephalopods more in detail, both as a class and in its two main lineages Decapodiformes and Octopodiformes, a phylogenetic tree was constructed. The C2H2 gene family underwent major expansions in cephalopods, evidenced by several big monophyletic groups in the resulting tree (here also referred to as expansion clusters) containing both squid and/or octopus sequences (Figure 2). In total, 19 expansion clusters met the paralog counting requirements (see STAR Methods, Figure S2; Table S3). The five largest, in terms of number of paralogs, account for over two-thirds of the expanded sequences and were lineage specific (e.g., octopus-specific cluster 4 with 108 sequences, Figure 2A). We characterized 11 squid-specific expansion clusters, containing mostly *D. pealeii* and *E. scolopes* sequences, with the biggest cluster containing 984 genes (Figure S2A, Data S1). 10 out of those 11 clusters showed a significant degree of confidence as their S-H support values were above the significant threshold (>0.7). The exception was squid cluster 8 with 36 sequences (Figure S2A, supplemental data). Four octopus-specific expansion clusters were identified containing between 104 and 495 sequences (Figure S2B), containing mostly *O. bimaculoides* genes. The biggest octopus cluster (495 sequences) included solely *O. bimaculoides* sequences but did not reach the threshold of significance (<0.7), meaning that there is little signal in the dataset to support this expansion. Furthermore, four smaller cephalopod-specific clusters were found (Figure S2C), containing 163, 132, 76, and 39 sequences. Three of the four cephalopod clusters had S-H support values greater than 0.7, with cephalopod cluster 1 (163 sequences) being the exception in that group (Figure S2C).

### C2H2 duplicated genes in cephalopods maintain the ancestral brain expression domain but show increased expression intensity

To gain insights about the function of C2H2 zinc fingers in cephalopods and explore the differences between conserved and duplicated genes, expression values of C2H2 genes were analyzed in each species individually (i.e., *E. scolopes*, *O. bimaculoides*, *O. vulgaris*, and *C. minor*). In *E. scolopes* (Figure S3), *O. bimaculoides* (Figure S4), and *O. vulgaris* (Figure S5), the relative expression mean of all C2H2 genes was found highest in the brain, followed by neural tissues distributed in the periphery (e.g., retina/eyes, skin, gastric ganglion, and axial nerve cord). However, in the two octopus’ species, which were the only ones with expression data available for each of the three central brain masses, the subesophageal one showed a lower relative expression mean compared to peripheral nervous tissues. Moreover, in *C. minor*, the whole central brain had the second highest relative expression mean, following the ovary (Figure S6).

**Figure 3:**
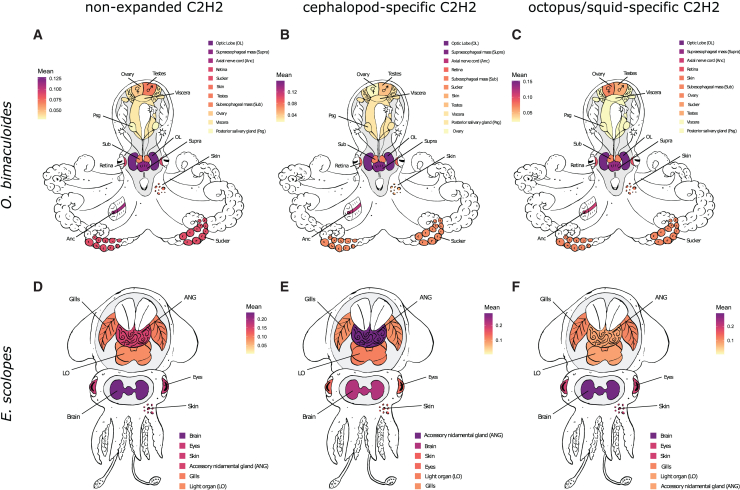
Relative C2H2 tissue expression in *E. scolopes* and *O. bimaculoides* (A–C) Expression of (A) non-expanded, (B) cephalopod-specific expanded, and (C) octopus-specific expanded C2H2s in *O. bimaculoides*. (D–F) Expression of (D) non-expanded, (E) cephalopod-specific expanded, and (F) squid-specific expanded C2H2s in *E. scolopes*. Tissues in legend are sorted from highest gene expression (top) to lowest gene expression (bottom). Colors are taken from corresponding boxplots in Figures S3 and S4.

To further investigate putative functional transitions between the different expansion stages, the relative expression of expanded and non-expanded C2H2 genes was analyzed separately in each species. In *E. scolopes*, the highest expression of non-expanded and cephalopod-specific C2H2s was found in the brain and the accessory nidamental gland (Figures 3D and 3E). For the squid-specific expanded genes, the brain remained the tissue with the highest relative expression but followed by the eyes (Figure 3F). In *O. bimaculoides*, the three groups (i.e., non-expanded, cephalopod, and octopus expansions) showed similar trends, where the optic lobe, the supraesophageal mass, and the axial nerve cord had the highest mean (Figures 3A–3C). The relative expression of non-expanded and cephalopod-specific C2H2s in *O. vulgaris* was highest in the optic lobe, the supraesophageal mass, and the arm tip (Figure S5). By comparison, the tissues with highest mean of relative expression values in the octopus-specific expansion group were the arm tip and the gastric ganglion, followed by the optic lobe and supraesophageal mass. In *C. minor*, the tissues with the highest expression in the non-expanded group were the ovary, intestine, and testis (Figure S6). The brain was the fourth tissue in this group of genes with highest mean relative expression. Lastly, both cephalopod- and octopus-expanded genes showed the highest relative expression values in the ovary and brain.

Absolute expression values were also compared statistically between expansion groups but in each tissue (Figures S7–10), to investigate if differences could be found in gene expression intensity. In *E. scolopes* (Figure S7), the cephalopod-expanded C2H2s had the highest absolute expression mean in all tissues. However, significant differences (Data S1) were only found between cephalopod- and squid-specific C2H2s in the gills, skin, and light organ (the latter also significantly different between cephalopod- and non-expanded C2H2s) and between all three groups in the accessory nidamental gland. The absolute expression in *O. bimaculoides* (Figure S8) was significantly higher in the cephalopod-specific expanded C2H2 genes in all but the ovary tissues. These differences were found to be significant between cephalopod-specific and non-expanded C2H2s as well as between cephalopod- and octopus-specific expanded C2H2s in six of the 11 tissues (i.e., optic lobe, supraesophageal mass, axial nerve cord, retina, skin, and subesophageal mass). In four tissues (i.e., suckers, testes, viscera, and posterior salivary gland), significant differences between all three groups were found. In the ovaries, the expression of octopus-specific expansions was found to be significantly higher than the other two groups. In the absolute expression of *O. vulgaris* (Figure S9), there were only significant differences in two of 9 tissues (i.e., the optic lobe and the supraesophageal mass), where again the cephalopod-specific expansion was higher than the non-expanded and octopus-specific groups. Despite the lack of statistical significance, in all tissues, the cephalopod-specific C2H2s followed the trend in the aforementioned species, with the highest absolute expression mean of all three groups. Lastly, *C. minor* (Figure S10) cephalopod-specific expanded C2H2 genes were also found to be expressed significantly higher in all tissues in comparison to all other expansion groups (only in the liver, the significant difference was between the cephalopod-specific and the non-expanded genes).

Altogether, no considerable differences in tissue expression patterns were found between non-expanded, cephalopod-specific, and squid-/octopus-specific expanded C2H2s in all four cephalopod species, as the highest relative expression mean was generally found in central brain tissues (Figures S3–S6 and 3). However, in all four species analyzed, the expression of C2H2 genes found in cephalopod-specific expansions was consistently higher than the more conserved and octopus/squid-expanded genes across all tissues.

### Co-expression analysis highlights the close association of expanded C2H2 genes and specific nervous system-related modules

**Figure 4:**
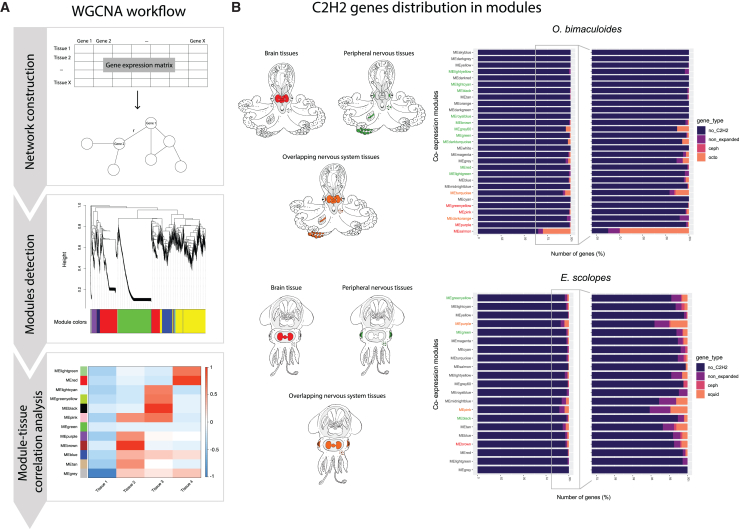
Co-expression analysis with WGCNA and distribution of C2H2 genes in the resulting modules in *O. bimaculoides* and *E. scolopes* (A) Example of a weighted gene correlation network for each species constructed using a gene expression matrix. The edge weights linking the different nodes (genes) are calculated based on the correlation coefficient (r) between gene expression patterns. Modules are then detected using hierarchical clustering and named after colors (e.g., MEgreen and MEred). Lastly, correlation between tissues and module eigengenes (i.e., the first principal component of each module) was calculated. (B) Distribution of C2H2 genes in the resulting modules in *O. bimaculoides* and *E. scolopes*, shown as percentage of gene numbers (from 0% to 100% in left barplot and zoom-in to a shorter range in right barplot, for greater detail). Module names are color-coded based on their correlation to nervous tissues: red for brain tissues, green for peripheral nervous tissues, and orange for overlapping central and peripheral nervous tissues. Modules colored black were not specifically correlated to nervous tissues.

Gene co-expression modules were constructed using weighted gene co-expression network analysis (WGCNA)^20^^,^^21^ (Figure 4A) to further investigate the putative functional correlation between different C2H2 genes (e.g., non-expanded and expanded) and between C2H2s and other expanded gene families (e.g., GPCRs). The analyses performed for the four species with adult tissue expression data available resulted in 21 modules for *E. scolopes*, 29 for *O. bimaculoides*, 45 for *C. minor*, and 75 for *O. vulgaris* (available from the Dryad Digital Repository, https://doi.org/10.5061/dryad.jh9w0vtfb).

The distribution of all C2H2s in the resulting co-expression modules was not found to be specific to modules uniquely correlated to nervous system tissues (Figures 4B and S11–S16). This was particularly true for *E. scolopes*, where only 1 module (i.e., MEgrey, a module with expression in all tissues but the ANG, Figure S13) did not contain any C2H2 genes (Figure 4B). Additionally, the modules showed a generally low percentage of C2H2s within them (i.e., number of C2H2s per total number of genes in the module), independent of their expansion state. More specifically, in all four species, this percentage was found to be below 10% (Figures 4B, S11, and S12). The only exception was the brain-related module MEsalmon in *O. bimaculoides* with 35% of C2H2s (Figures 4B and S14). This outlier case could well be related to the higher number of C2H2 genes found in this species in comparison to the other three ones (i.e., 1,453 in *O. bimaculoides* versus 809, 228, and 280 in *E. scolopes*, *O. vulgaris*, and *C. minor*, respectively, Figure S1).

Despite the lack of a clear association of C2H2s with nervous system-specific modules, some of these modules showed the highest percentages of expanded C2H2 genes. In *O. bimaculoides*, we found that MEsalmon (brain-related) and MEturquoise (related to both central and peripheral nervous tissues) showed the two highest percentages of C2H2s, particularly genes from octopus-specific expansions (Figures 4B and S14). In *E. scolopes*, MEpink and MEpurple (both related to central and peripheral nervous tissues) showed the two highest counts of C2H2 genes and, specifically, squid-specific expanded genes (Figures 4B and S13). Moreover, in *O. vulgaris*, we identify MEgreen, MEmagenta, and MEturquoise, as brain-related modules (Figure S15). MEmagenta showed the highest percentage of octopus-specific expanded genes, and cephalopod-expanded C2H2s were highest in MEgreen and MEturquoise (Figure S11). Lastly, nervous system-specific modules in *C. minor* containing most cephalopod-expanded C2H2s were the brain-related modules MEbrown, MEturquoise, and MEyellow (Figures S12 and S16). MEbrown and MEyellow contained additional octopus-specific expanded genes.

**Figure 5:**
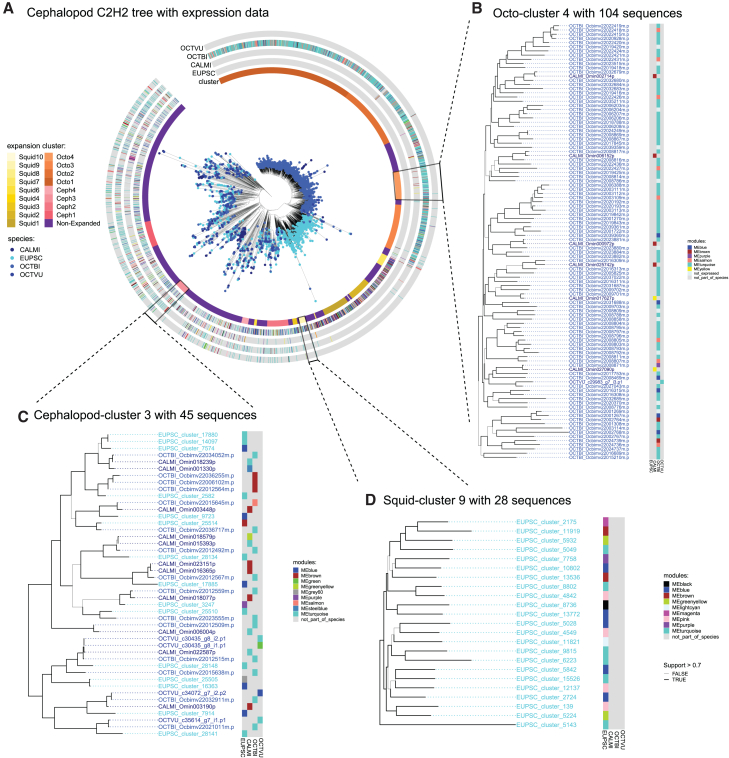
Trimmed tree containing only C2H2 genes of *E. scolopes*, *C. minor*, *O. bimaculoides*, and *O. vulgaris* including information on the corresponding co-expression modules (A) Tree with cephalopod C2H2 sequences with expression data. The tree was trimmed to only contain sequences from cephalopod species with expression data, namely *E. scolopes*, *C. minor*, *O. bimaculoides*, and *O. vulgaris.* Therefore, zoom-ins on the same clusters as in Figure 2 may contain fewer sequences, if it also holds sequences from a cephalopod species without expression data. Annotation columns give additional information on the co-expression module and the expansion state (cephalopod-specific expansions marked in pink tones, squid-specific expansions marked in yellow tones, octopus-specific expansions marked in orange tones, and non-expanded/conserved C2H2s marked in purple). (B) Zoom-in on an octopus-specific expansion cluster. (C) Zoom-in on a cephalopod-specific expansion cluster. (D) Zoom-in on a squid-specific expansion cluster.

To further investigate the evolutionary relationship of the modules and explore if genes of the same expanded group belonged to the same co-expression module, a phylogenetic tree with only the cephalopod C2H2 genes and their corresponding module was visualized (Figure 5). However, no particular co-expression module corresponded to an expansion group; rather, paralogs belonged to different modules. Altogether, modules with the highest number of expanded C2H2 genes were found to be specifically related to nervous system tissues, but these expression modules are not defined by evolutionary relationships.

### Enriched C2H2 modules show putative functional associations with other neuronal gene families

To investigate putative regulatory associations of C2H2 genes in relation to other expanded gene families like the GPCRs, a GO term analysis was performed in the three modules with highest number of C2H2 genes in all four species (Table S4). These results showed that the most abundant biological process GO terms were: “GO:0007186 – G protein-coupled receptor signaling pathway” (6 out of 12 modules, Table S4), “GO:0006511 – ubiquitin-dependent protein catabolic process” (4 out of 12 modules), “GO:0006886 – intracellular protein transport” (4 out of 12 modules), and “GO:0006357 – regulation of transcription by RNA polymerase II” (4 out of 12 modules). An additional analysis of the molecular function ontology was done as a control step to identify modules with enriched C2H2 GO terms. In 7 out of 12 modules, significant enrichment of the main C2H2 GO term “GO:0003676 – nucleic acid binding” was found. Those modules were most of the ones highlighted previously in the previous results section, namely MEsalmon and MEturquoise in *O. bimaculoides*, MEpink and MEpurple in *E. scolopes*, MEbrown and MEturquoise in *C. minor*, and MEturquoise in *O. vulgaris*, all of which are either brain or overlapping nervous system modules. Out of the 12 modules with the highest percentages of expanded C2H2s, 2 were found to have enrichments of both C2H2 and GPCR GO terms (i.e., *E. scolopes* MEpurple and *O. vulgaris* MEturquoise), comprising 25 cephalopod-specific and 145 lineage-specific expanded C2H2 genes (Table S4).

## Discussion

While previous studies were able to show general association in C2H2 expression with neural tissues in cephalopods^7^^,^^8^ and strong co-localization of the genes in just a few chromosomes,^17^ the evolutionary timing and further dissection of their expression patterns remained elusive due to the lack of expression data and genomic sequences of other coleoids. With the inclusion of various squid and octopus’ species genomes, we reconstructed expansions of this gene family at different evolutionary periods, distinguishing ancient proteins shared with other metazoans from genes that duplicated during the coleoid radiation. Altogether, our analyses shed light on the evolutionary history and expression dynamics of one of the largest coleoid cephalopod gene families—the zinc finger type C2H2 transcription factors.

### Expanded C2H2s in cephalopods maintained their nervous system-related expression

In total, 16 expansion events were found with our phylogenetic approach. The majority of these expansions (i.e., 13 out of 16) originated after the Decapodiformes/Octopodiformes split, while only three emerged in cephalopods before the separation of these major coleoid lineages. Our results showed differences in C2H2 numbers between some cephalopod species, which could be explained by missing paralogs resulting from the different available gene predictions of each genome. Nevertheless, the expansion analysis, given the total size of the gene family, should not be affected by potentially missed or mispredicted genes.

Studying the expression of this gene family, we confirmed previous findings^7^^,^^8^ that C2H2s generally are expressed in nervous tissue. However, the contribution of this gene family differed between the various central brain lobes. In those species with data in hand, namely *O. bimaculoides* and *O. vulgaris*, the contribution of C2H2s was highest in the optic lobe and supraesophageal mass, while the subesophageal mass showed a lower expression than peripheral nervous tissues.

Moreover, no clear shift in tissue expression pattern was found between expansion groups (Figures 3 and S1–S4). This result contrasts with the findings on the GPCR gene family expansion in cephalopods, where expanded genes were expressed in other tissues outside of the brain and conserved genes were consistently expressed in the brain.^9^ For instance, some of the GPCR duplicates were expressed in the axial nerve cord, the eyes, the skin, and the gills. Despite the lack of tissue expression shift, we found a consistent difference between expanded and non-expanded genes in expression levels, as cephalopod-specific C2H2 expression was significantly higher than that in non-expanded and octopus-/squid-specific genes in 31 of 45 tissues. Such trends will be interesting to investigate further in the context of neuronal networks where C2H2s are particularly enriched. It is possible that any functional significance of C2H2 expansion may be related to the expansion of the central and peripheral cephalopod nervous system and the associated increase in number of neurons. Further investigation of the expanded genes by, e.g., examining the annotation of non-cephalopod sequences of their sister groups in the phylogenetic tree and investigating their expression in other species would also help to better understand the commonalities behind C2H2 zinc finger expansions and contribution to the evolution of nervous systems in metazoans.

### Paralogs are present in different gene expression modules suggesting a change in regulation

While overall C2H2 expression remains in the domains of the neuronal tissues and mainly the central brain, further analysis using WGCNA revealed that expanded genes have contributed to different co-expression modules. Even within the same expansion clade, C2H2 genes did not share the same module identity (e.g., Figure 5A). Our prediction is thus that, despite the observation that gene duplicates can occur in tandem^15^ and may share regulatory elements, C2H2 duplications in cephalopods have resulted in a switch in their regulation such that duplicates are often associated with different expression modules. This may be a reflection of selective pressure to diversify regulation that is potentially associated with different neuronal cell types following gene duplication. WGCNA has been previously used to investigate gene regulatory networks in different biological contexts,^22^ but further analyses would be needed to test our prediction. For instance, because the expression data analyzed here correspond to whole tissues containing different cell types from independent experimental sources, leveraging single-cell transcriptome data from cephalopod brains may provide fruitful insights into the nature of the regulation of these C2H2 module identities.

### Contrast to GPCRs—Complementary evolutionary paths between gene family expansions in cephalopods

**Figure 6:**
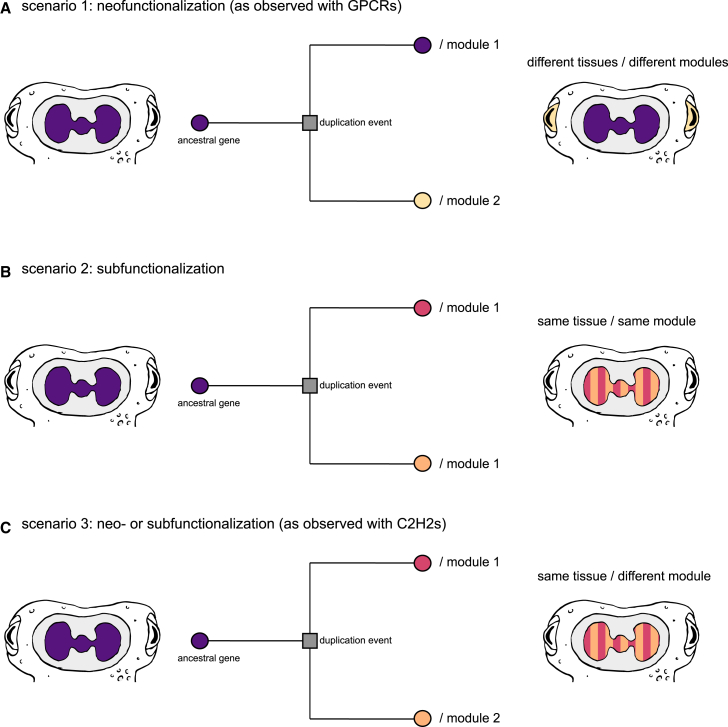
Potential evolutionary scenarios of duplicated genes (A) scenario 1 represents the classical neofunctionalization hypothesis, where one of the duplicates keeps the ancestral function, while the other gains a new function and is expressed in a different tissue, therefore being allocated to different expression modules. This was observed, e.g., in cephalopod GPCRs. (B) scenario 2 represents the classical subfunctionalization hypothesis, where the duplicates divide the ancestral function while maintaining expression in the same tissue and are allocated as well in the same co-expression module. (C) scenario 3 represents the results as observed in this study with cephalopod C2H2s, where the duplicates remain mostly expressed in the same tissue as the conserved genes but are allocated to different co-expression modules.

The paralogous genes of the two largest gene family expansions in cephalopods—C2H2 and GPCRs—have contributed to different tissues. On one hand, expanded GPCRs shifted to expression domains outside the brain possibly through neofunctionalization^9^ (Figure 6A). On the other hand, this study found that C2H2s maintained a central nervous tissue expression. However, this did not imply the preservation of co-expression module identity between paralogous genes (Figure 6B) but rather a divergence into different modules (Figure 6C). These two gene families share the timing of some expansions, as both GPCRs and C2H2s were characterized by cephalopod- and octopus-specific expansions. This combination of shared expansion timing and differences in expression patterns of expanded genes in both families may suggest a functional link between sensory field expansion in the peripheral nervous system and neuronal diversity. This hypothesis is not contradicted by the results of the GO term analysis, where only 2 out of 12 tested modules showed enrichment for both C2H2s and GPCRs. This can be explained by the vastly different direction of expansion in those two gene families. The analysis was focused on C2H2-enriched modules, which were mostly brain related, but the majority of GPCR expansions can only be found outside of the brain, therefore indicating a more complex underlying connection. Both C2H2 signatures of brain modules and their potential evolutionarily coupled GPCR expansions may thus provide cues for when certain circuits have emerged.

### Evolution of C2H2-binding sites and cephalopod genome expansions

Several studies have proposed that C2H2 and repetitive element expansions are often correlated, with certain repetitive sequences showing propensity to become co-opted as binding sites for C2H2.^15^ In general, this binding activity has been shown to drive the silencing of transposable element loci via DNA methylation, with genome-wide impact on gene regulatory network evolution.^23^ How much the repeat-rich and, compared to non-cephalopod mollusks, expanded genomes of coleoid cephalopods^7^^,^^24^ may have formed the ground for the evolutionary arms race between transposon accumulation and C2H2-enabled silencing is now an open question. So far, only few ancient repeat element expansions, shared between and dating back to the last common ancestor of the Octopodiformes and Decapodiformes lineages, have been identified.^24^ The vast majority of repeat content in coleoid cephalopod genomes is lineage specific, i.e., short interspersed nuclear elements are more abundant in octopus genomes, whereas large interspersed nuclear elements are more prevalent in the squid genomes. While going beyond the scope of this study, the next steps would consist of identifying the recently expanded repeat elements that may harbor binding sites for some of the recently duplicated C2H2 families and how these binding sites may differ between octopuses and squids.

### Closing remarks

Our study provides a comprehensive phylogenetic and expression analysis of C2H2 zinc fingers in cephalopods, building on previous findings for these large families.^25^ We characterized expansions that occurred in cephalopods at Decapodiformes (i.e., squid) and Octopodiformes (i.e., octopus) phylogenetic level. We found that the expanded C2H2 genes maintained the ancestral expression domain: the nervous system and mainly the brain. This result contrasts with previous findings in other expanded gene families, like the GPCRs, where paralogs shifted their expression patterns from the central brain to the peripheral nervous system and non-nervous tissues (Figure 6A). Moreover, further analyses showed us that expanded genes originating from the same expansion events do not belong necessarily to the same co-expression modules (Figure 6C), suggesting that the functional divergence of paralogs might be related to changes in regulatory processes within the same tissue rather than to the gain of an entirely new function elsewhere. The diversification of recently duplicated C2H2s among distinct expression modules proposes a testable hypothesis of functional diversification, either through neo- or subfunctionalization (Figure 6C). These results set the stage for further dissection of this large gene family, and additional data, in particular from single-cell and spatial transcriptomics experiments, will help test these hypotheses and the evolutionary and functional relationship to other gene family expansions. Finally, the data presented here on the largely neuronally expressed cephalopod-expanded C2H2s may help set the stage for the further identification of the evolutionary forces that drive the evolutionary expansion trends of this family in metazoans, including but not limited to co-option and co-expansion of other gene families as well as repetitive elements and C2H2-binding sites.

### Limitations of the study

The expression analyses rely on experiments performed in independent studies and with different tissue samplings, limiting the extension of conclusions that can be drawn from between-species comparisons. Moreover, our results provide insights into putative regulatory correlations between expanded genes in cephalopods, but since these come from gene expression in whole tissues containing a variety of cell types, further studies would benefit from expression data with single-cell resolution and/or spatial transcriptomics.

## Resource availability

### Lead contact

Requests for further information and resources should be directed to and will be fulfilled by the lead contact, Oleg Simakov (oleg.simakov@univie.ac.at).

### Materials availability

This study did not generate new unique reagents.

### Data and code availability

Data are available under https://doi.org/10.5061/dryad.jh9w0vtfb. Code is available under https://bitbucket.org/viemet/public/src/master/C2H2/.

## Acknowledgments

This research (C.H., E.A.R., and O.S.) was funded by the Austrian Science Fund (10.13039/501100002428FWF) grant P30686-B29 (https://doi.org/10.55776/P30686). Computation was done using the Life Sciences Cluster at the 10.13039/501100003065University of Vienna. The Stazione Zoologica Anton Dohrn (Italy) co-financed E.A.R. PhD. C.B.A. acknowledges generous support through the MBL Early Career Fellows gift from Susan and David Hibbitt.

## Author contributions

C.H., E.A.R., and O.S. designed the study. C.H. and E.A.R. conducted the analyses and wrote the manuscript, with input from all co-authors.

## Declaration of interests

The authors declare no competing interests.

## STAR★Methods

### Key resources table

**Table:** 

REAGENT or RESOURCE	SOURCE	IDENTIFIER
**Deposited data**		

Phylogenetic analysis	this study	https://doi.org/10.5061/dryad.jh9w0vtfb
Expression module analysis	this study	https://doi.org/10.5061/dryad.jh9w0vtfb

**Software and algorithms**		

MAFFT	Katoh et al.,^26^	NA
IQTREE	Minh et al.,^27^	NA
FigTree	available online^28^	NA
WGCNA	Langfelder et al.,^29^	NA

### Experimental model and study participant details

No experiments were conducted in this study and the underlying data was collected from publicly available genomes (Table S1).

### Method details

#### Data acquisition and tree construction

A dataset of protein sequences for the C2H2 zinc finger gene repertoire of 20 animal species was compiled from various sources (Table S1). The species were chosen to cover a wide evolutionary range containing protostomes and deuterostomes, thus enabling a broad cross-phyla comparison. Seven of the 20 species belonged to the coleoid cephalopods; namely *Architeuthis dux* (giant squid), *Callistoctopus minor* (long arm octopus), *Doryteuthis pealeii* (longfin inshore squid), *Euprymna scolopes* (Hawaiian bobtail squid), *Idiosepius paradoxus* (northern pygmy squid), *Octopus bimaculoides* (California two-spot octopus) and *O. vulgaris* (common octopus). The remaining 13 species were *Anopheles gambiae* (African malaria mosquito), *Branchiostoma floridae* (Florida lancelet), *Caenorhabditis elegans* (roundworm), *Capitella teleta* (polychaete worm), *Crassostrea gigas* (Pacific oyster), *Drosophila melanogaster* (fruit fly), *Helobdella robusta* (Californian leech), *Homo sapiens* (human), *Lottia gigantea* (giant owl limpet), *Mizuhopecten yessoensis* (Japanese scallop), *Saccoglossus kowalevskii* (acorn worm), *Schistosoma mansoni* (blood fluke) and *Strongylocentrotus purpuratus* (purple sea urchin).

The sequences for the non-cephalopod species were gathered from the UniProt database using the Pfam C2H2 zinc finger domain ID (PF00096). If possible, only sequences from an UniProt proteome were used (Table S1). Cephalopod whole-genome protein sequences were taken from published data^7^^,^^8^^,^^17^^,^^30^^,^^31^^,^^32^ (Table S1) and scanned against InterPro signatures using InterProScan.^23^ Only sequences that matched the signature PF00096 were selected. The FASTA files were filtered by isoforms to only contain the longest protein sequence per gene. Sequences were then aligned using MAFFT (v7.427) and subsequently trimmed using trimAl (v1.4.rev15) with a gap threshold of 0.25, a residue overlap of 0.25 and a sequence overlap of 90%. To find the right model of molecular evolution for the Maximum-likelihood tree construction, the built-in IQTREE (v1.6.2) method ModelFinder was used and resulted in the Whelan and Goldman model (WAG) to be the best fitting amino acid replacement model for the dataset. The tree was then constructed with FastTree (v2.1.10) and re-rooted manually in FigTree (v1.4.4). To know the confidence degree of the resulting topology, branch support values were computed by default with FastTree using the Shimodaira-Hasegawa (S-H) test, which compares multiple topologies based on a non-parametric bootstrap.^33^^,^^34^ The significance threshold of S-H support values here used was 0.7. The largest monophyletic cluster containing only *B. floridae* sequences (314 in total) was used as an outgroup, because this animal represents the most evolutionarily distant species to the cephalopods from the taxa sampled for our phylogenetic analysis. For tree visualization the R package ggtree^35^ was used.

#### Identification of cephalopod expansions

A list of expansion clusters was created, defined as monophyletic groups containing only sequences from the seven cephalopod species of interest. Such clades represent putative gene expansions through duplication, because in a scenario of no duplications it should contain only one gene per species (i.e., only the orthologues). Depending on the species assemblage of the sequences within the cluster, these were classified as cephalopod-, squid- or octopus-specific expansions. Due to the lack of data availability of other cephalopod lineages, no further classifications were applied.

Following previous findings on the size of expansions in cephalopods (i.e., GPCRs^9^ and protocadherins^10^), monophyletic groups with at least 20 sequences solely belonging to either squid or octopus species were counted as Decapodiformes-specific expansions (or squid-specific) or Octopodiformes-specific expansions (or octopus-specific), respectively. However, if a cluster mostly contained sequences of one lineage (either Decapodiformes or Octopodiformes) and less than four sequences from the other lineage, it was still assigned as lineage-specific (i.e., squid-/octopus-specific). Lastly, clusters with at least 30 sequences belonging to both lineage groups (i.e., squid and octopus) were counted as cephalopod-specific expansions.

Following these classification parameters, all the C2H2 sequences analyzed were attributed to being either “non-expanded” (i.e., if not belonging to any expansion cluster) or “expanded” (i.e., if belonging to an expansion cluster, either cephalopod-, squid- or octopus-specific).

### Quantification and statistical analysis

#### Statistical analyses of tissue expression

Tissue expression data can help to gain insights into the putative function of genes, by narrowing down the possible cell types where their product, i.e., the protein, is needed. Thus, the expression of the C2H2 zinc fingers was investigated in cephalopod species with a published genome for which RNA-Seq data of different tissues (Table S2) was publicly available (i.e., *C. minor*,^31^ *E. scolopes*,^8^ *O. bimaculoides*^7^ and *O. vulgaris*^30^). Normalized expression data was used (transcripts per million or TPM^36^ in case of *C. minor* and *O. vulgaris*, copies per million or CPM^37^ in *O. bimaculoides* and trimmed mean of M values or TMM^38^ for *E. scolopes*) for downstream analyses and only comparisons within species were performed. All species, excepting *O. vulgaris,* had one sample per tissue. Because the *O. vulgaris* dataset had three replicates per tissue, the dataset was merged first previous to any of the following analyses by taking the median values of the replicates.

Expression values between non-expanded C2H2s, cephalopod-specific expanded C2H2s and lineage-specific expanded C2H2s (i.e., octopus- or squid-specific, depending on the species at hand) were firstly compared. To test for significant differences between the expression of these three different C2H2 groups in each tissue, a Levene’s test^39^ and the non-parametric Fligner-Killeen test^40^ were performed to assess normal distribution and variance homogeneity of the data. Because only seven out of 45 tissue datasets exhibited equal variances, the non-parametric test Kruskal-Wallis^41^ was used. For datasets with a significant *p*-value in the Kruskal-Wallis test, a Dunn’s test^42^ using the Bonferroni *p*-value adjustment for multiple comparison was done to check which of the three C2H2 groups showed the significant differences. Additionally, expression values for each gene were normalized by dividing its TPM/CPM/TMM count in a tissue with its maximal value. The resulting relative expression values, ranging from 0 to 1, were then used to visualize the expression trends of each C2H2 group (i.e., non-expanded, cephalopod-specific and octopus-/squid-specific) using boxplots, sorting these from lowest to highest group mean.

The statistical analyses and boxplots were done in R (v.3.6.1) using the packages ggplot2,^43^ dunn.test^44^ and car.^45^

#### Identification of gene co-expression modules

To further explore the correlation of expression patterns between the different C2H2s expansion groups and other gene families, a weighted gene co-expression network analysis was performed using WGCNA^20^^,^^21^ in R. First, the normalized expression tables of *E. scolopes, O. bimaculoides,* and *C. minor* were log-transformed to reduce skewness of the data toward large values. The same log-transformation was performed on the merged *O. vulgaris* dataset. The clustering analysis was performed block-wise with the *blockwiseModules* function with a minimal module size of 20 genes and using a signed co-expression measure (i.e., network with correlation values ranging from −1, meaning negative correlation, to 1, meaning positive correlation). As suggested by the developers (*33, 34*) for a signed network with less than 20 samples, the soft threshold power used was 18. Module names were assigned by WGCNA automatically using colour names (e.g., MEpink, MEpurple) and the genes found within each resulting co-expression module can be found in Supplementary Data. To summarize the clustering results, module-trait relationships were drawn by correlating the eigengenes, understood as the average expression profiles of the resulting modules, with each adult tissue. The correlation values were finally displayed in heatmaps.

The distribution of C2H2 genes in the resulting co-expression modules was explored with bar charts using the R package ggplot2.^43^ Only modules with a positive correlation to the brain or other nervous tissues were further examined. We defined brain-related modules as those with a significant correlation coefficient higher than 0.7 to one brain-related tissue (i.e., subesophageal-, supraesophageal mass, optic lobes or the brain as a whole tissue). Because in some species expression data was gathered from the different brain regions separately, some modules were correlated to more than one of those separate tissues (i.e., supraesophagael-, subesophageal mass and optic lobe). This resulted in lower correlation coefficients to brain-related tissues despite a putative generalized brain function of the genes. Thus, modules were also identified as brain-related if they showed a correlation coefficient greater than 0.3 exclusively with two or more brain-related tissues. Furthermore, brain-related modules were split into three groups: (i) modules solely expressed in brain tissues (whole brain, optic lobes, supraesophageal-, subesophageal mass) were referred to as brain modules (in *O. vulgaris* we used the term central nervous tissues, as it included the gastric ganglion and stellate ganglia); (ii) modules solely connected to peripheral nervous tissues or tissues that are known to have a higher number of peripheral nerve cells (i.e., axial nerve cord, eyes, retina, skin, suckers and arm tip) were referred to as peripheral nervous system modules; and (iii) modules expressed in one or more of the aforementioned brain tissues as well as in one or more of the peripheral nervous tissues were referred to as overlapping nervous system modules. Lastly, the distribution of co-expression modules in the phylogenetic tree was explored, focusing on the expansion clades. To do so, a subset of the original tree containing only the expanded cephalopod C2H2 genes was extracted and visualized with ggtree, using the modules as annotation columns.

#### GO term analysis

A gene ontology (GO) enrichment analysis was performed to identify potential regulatory relationships of C2H2 zinc finger proteins with other expanded gene families in cephalopods. For each of the four species with expression data, the three modules with the highest percentage of expanded C2H2 genes were selected and checked for GO term enrichment.^46^ Briefly, this analysis calculated the (corrected) statistical significance of how often a GO term from the biological processes or “BF” ontology category occurred in that group of genes (i.e., co-expression module) in comparison to all other genes in the genome. The GO term entries of all genes were isolated from the InterProScan^47^ results (see section (a) Data acquisition and tree construction). The GO term enrichment analysis was then performed using the R package topGO.^46^ Only GO terms with a *p*-value smaller than 0.05 were kept for further inspection and comparison.

## Supplemental information

Document S1. Figures S1–S16 and Tables S1–S4

Data S1. Statistical significance (*p* values) associated with comparisons of expression values between expansion groups (non-expanded, cephalopod-expanded, and octopus/squid-expanded C2H2s) in each tissue for *E. scolopes* (EUPSC), *C. minor* (CALMI), and *O. bimacu*
