# Supplementary material for: Characterization of C2H2 superfamily expansions in cephalopods and their contribution to nervous system evolution
Source: iScience. 2025 Sep 12;28(10):113561. doi: 10.1016/j.isci.2025.113561 (PMC12513265; doi:10.1016/j.isci.2025.113561)
Supplement: Document S1. Figures S1–S16 and Tables S1–S4 [file mmc1.pdf]

iScience, Volume *28*

Supplemental information

Characterization of C2H2 superfamily expansions in cephalopods and their contribution to nervous system evolution

Christina Holzinger, Elena A. Ritschard, Pamela Imperadore, Giovanna Ponte, Caroline B. Albertin, Graziano Fiorito, and Oleg Simakov

Supplemental Information

1. Figures

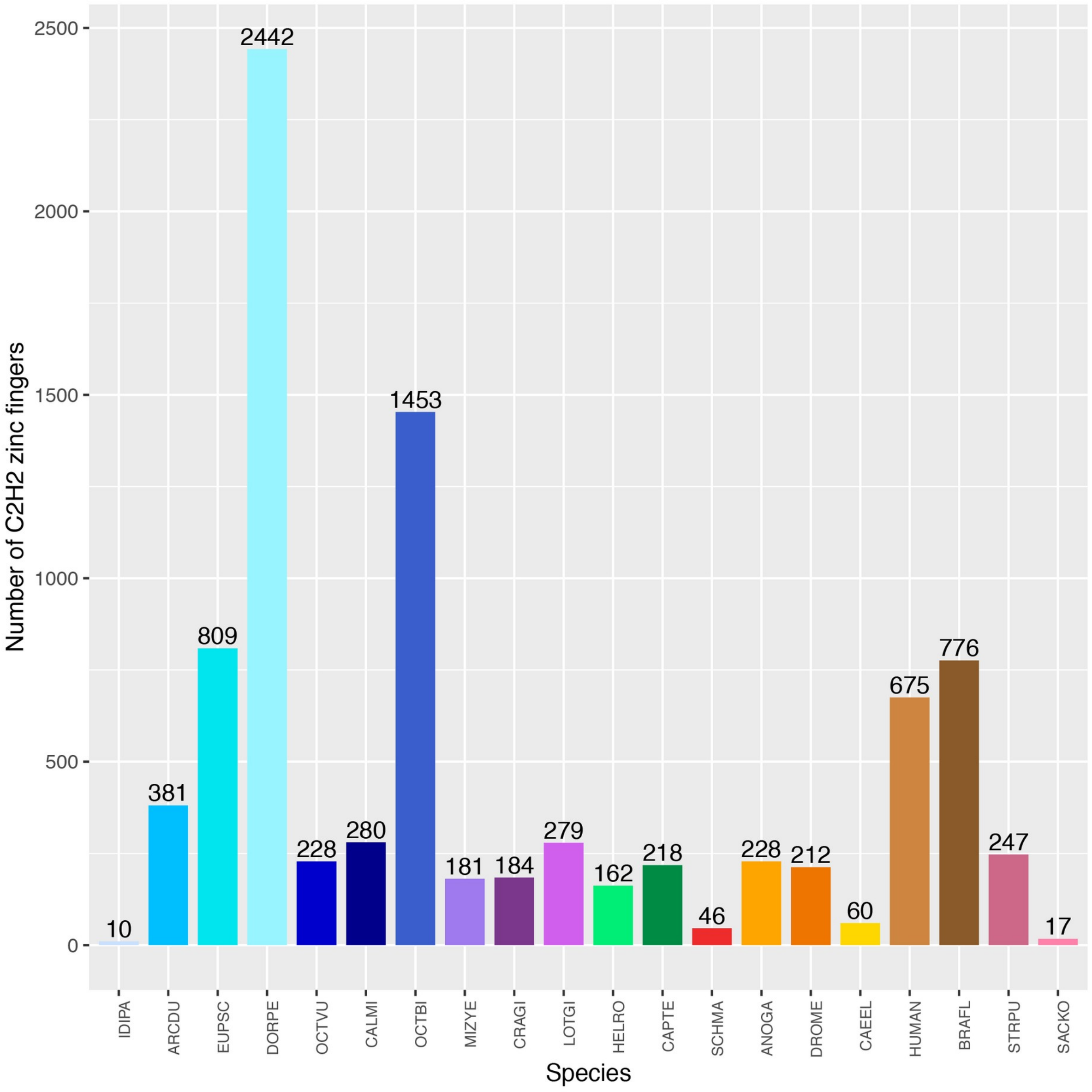

Figure S1. Number of C2H2 protein sequences per species used in tree construction. Species were sorted according to their evolutionary distance, with the four rightmost species (HUMAN, BRAFL, STRPU, SACKO) being deuterostomes and therefore acting as sister clade to the other protostome species, including the coleoid cephalopods (IDIPA, ARCDU, EUPSC, DORPE, OCTVU, CALMI, OCTBI). Abbreviations are as follows: ANOGA: *Anopheles gambiae*, ARCDU: *Architeuthis dux,* BRAFL: *Branchiostoma floridae,* CAEEL: *Caenorhabditis elegans*, CALMI: *Callistoctopus minor,* CAPTE: *Capitella teleta*, CRAGI: *Crassostrea gigas*, *DORPE: Doryteuthis pealeii,* DROME: *Drosophila melanogaster*, EUPSC: *Euprymna scolopes,* HELRO: *Helobdella robusta*, HUMAN, IDIPA: *Idiosepius paradoxus,* LOTGI: *Lottia gigantea*, MIZYE: *Mizuhopecten*

yessoensis, OCTBI: *Octopus bimaculoides*, OCTVU: *Octopus vulgaris*, SACKO: *Saccoglossus kowalevskii*, SCHMA: *Schistosoma mansoni* and STRPU: *Strongylocentrotus purpuratus.*

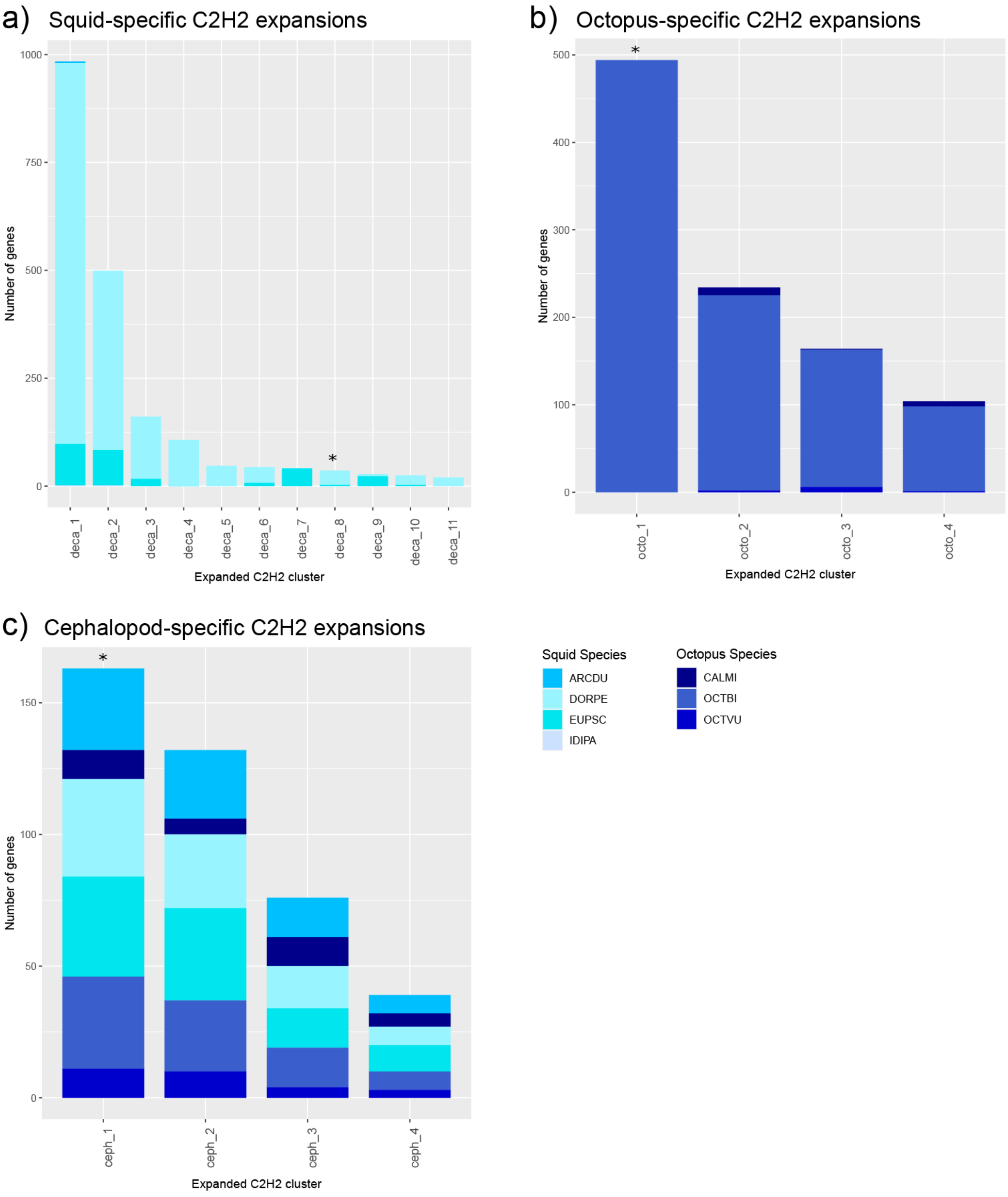

Figure S2. Number of genes per species in squid-, octopus- and cephalopod-specific C2H2 expansions. a) Squid-specific expansion clusters, ranging from 20 to 984 sequences. b) Octopus-specific expansion clusters, ranging from 104 to 495 sequences. c) Cephalopod-specific expansion clusters ranging from 39 to 163 sequences. All expansions had an SH support value greater than 0.7, with the exception of those marked with an asterisk (*). Abbreviations are as follows:

ARCDU: *Architeuthis dux,* CALMI: *Callistoctopus minor, DORPE: Doryteuthis pealeii,* EUPSC: *Euprymna scolopes,* IDIPA: *Idiosepius paradoxus,* OCTBI: *Octopus bimaculoides*, OCTVU: *Octopus vulgaris*.

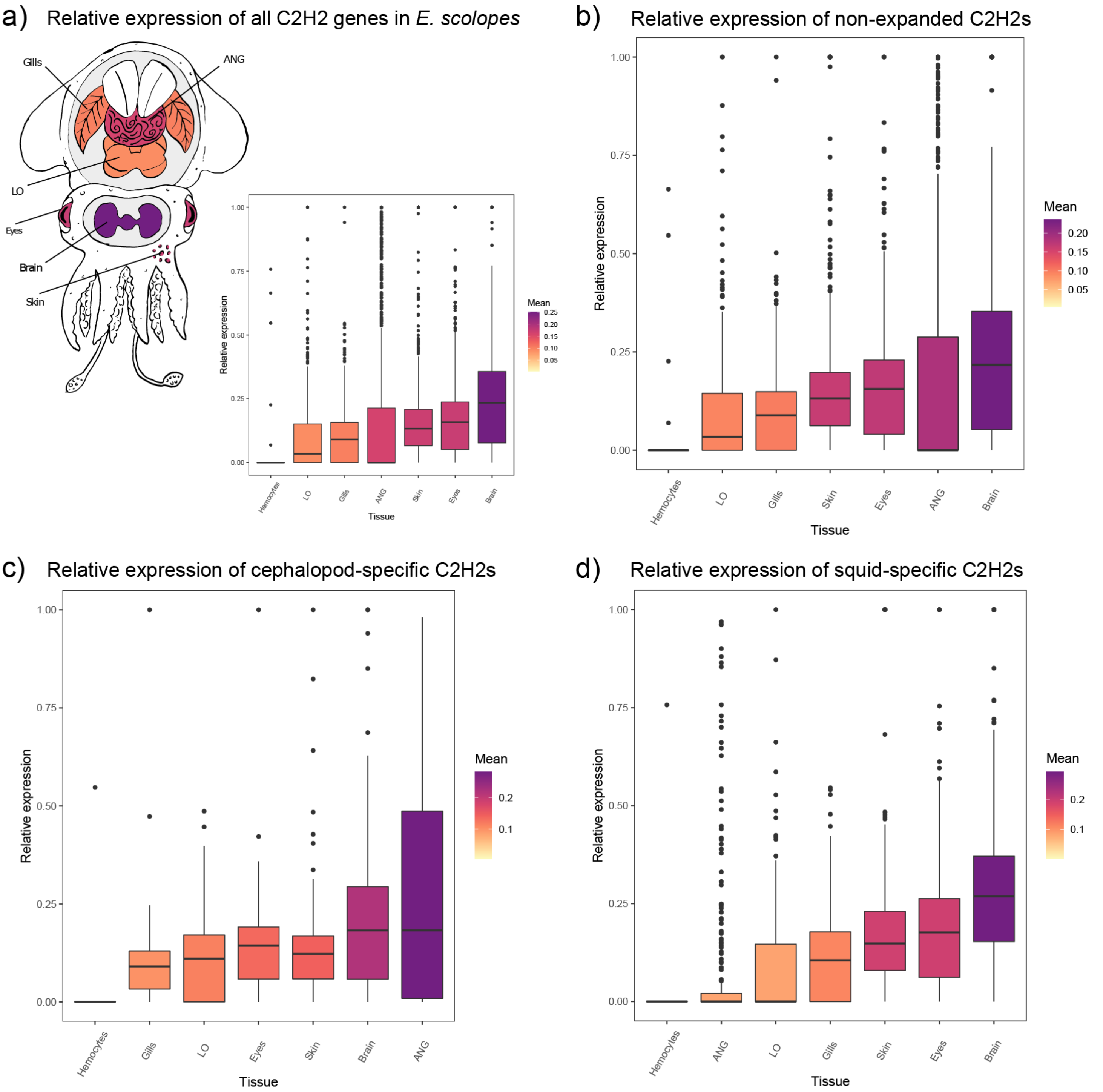

Figure S3. Relative expression of a) all C2H2 genes, b) non-expanded, c) cephalopod- and d) squid- specific expanded C2H2s in *E. scolopes*. Tissues in boxplots are sorted using their means, from lowest (left) to highest (right). The box colours were then used to colour the corresponding tissues in the animal sketches as in (a) and Figure 2d-f. Illustrations by Elena Ritschard.

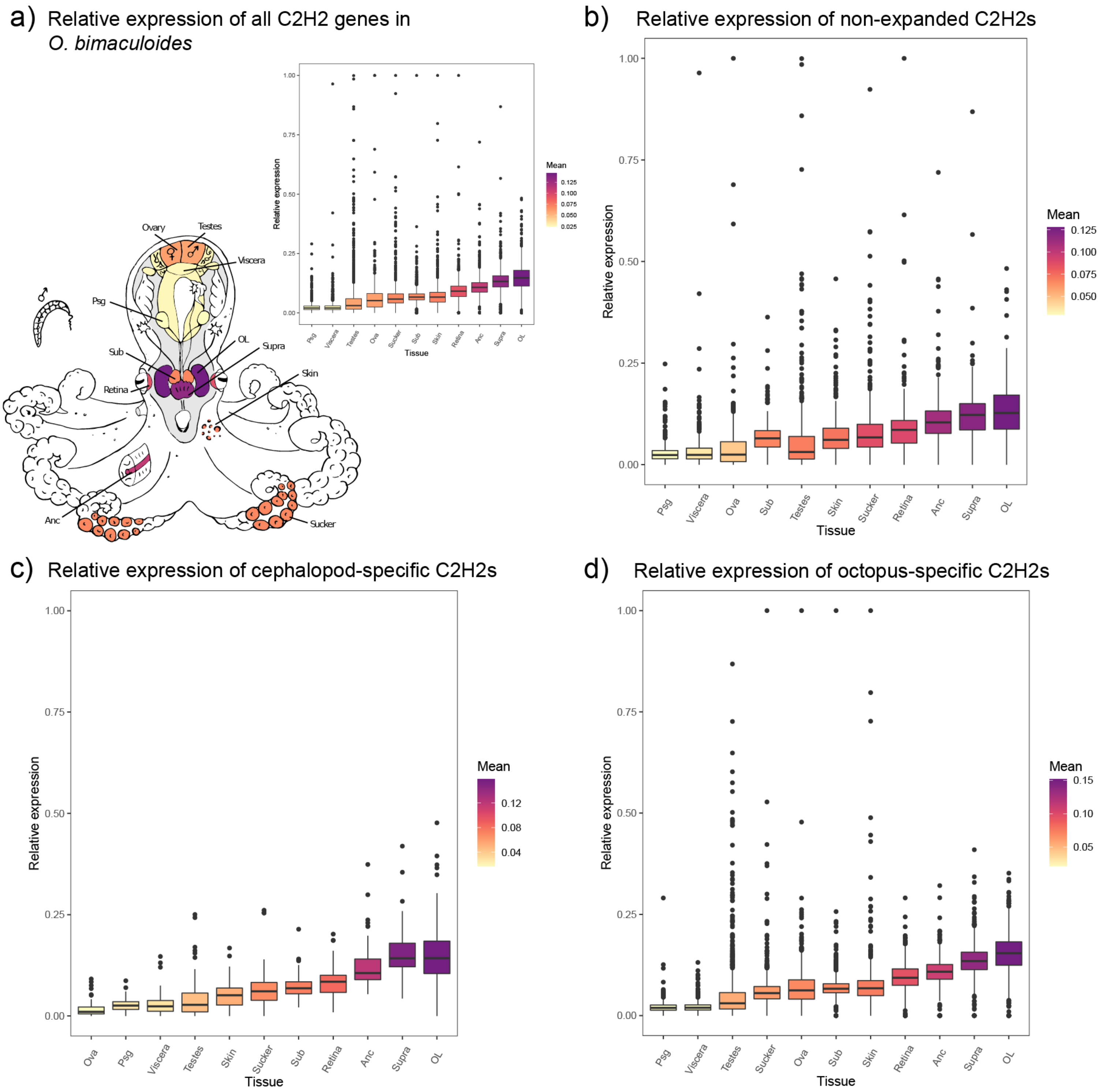

Figure S4. Relative expression of a) all C2H2 genes, b) non-expanded, c) cephalopod- and d) octopus- specific expanded C2H2s in *O. bimaculoides*. Tissues in boxplots are sorted using their means, from lowest (left) to highest (right). The box colours were then used to colour the corresponding tissues in the animal sketches as in (a) and Figure 2a-c. Illustrations by Elena Ritschard.

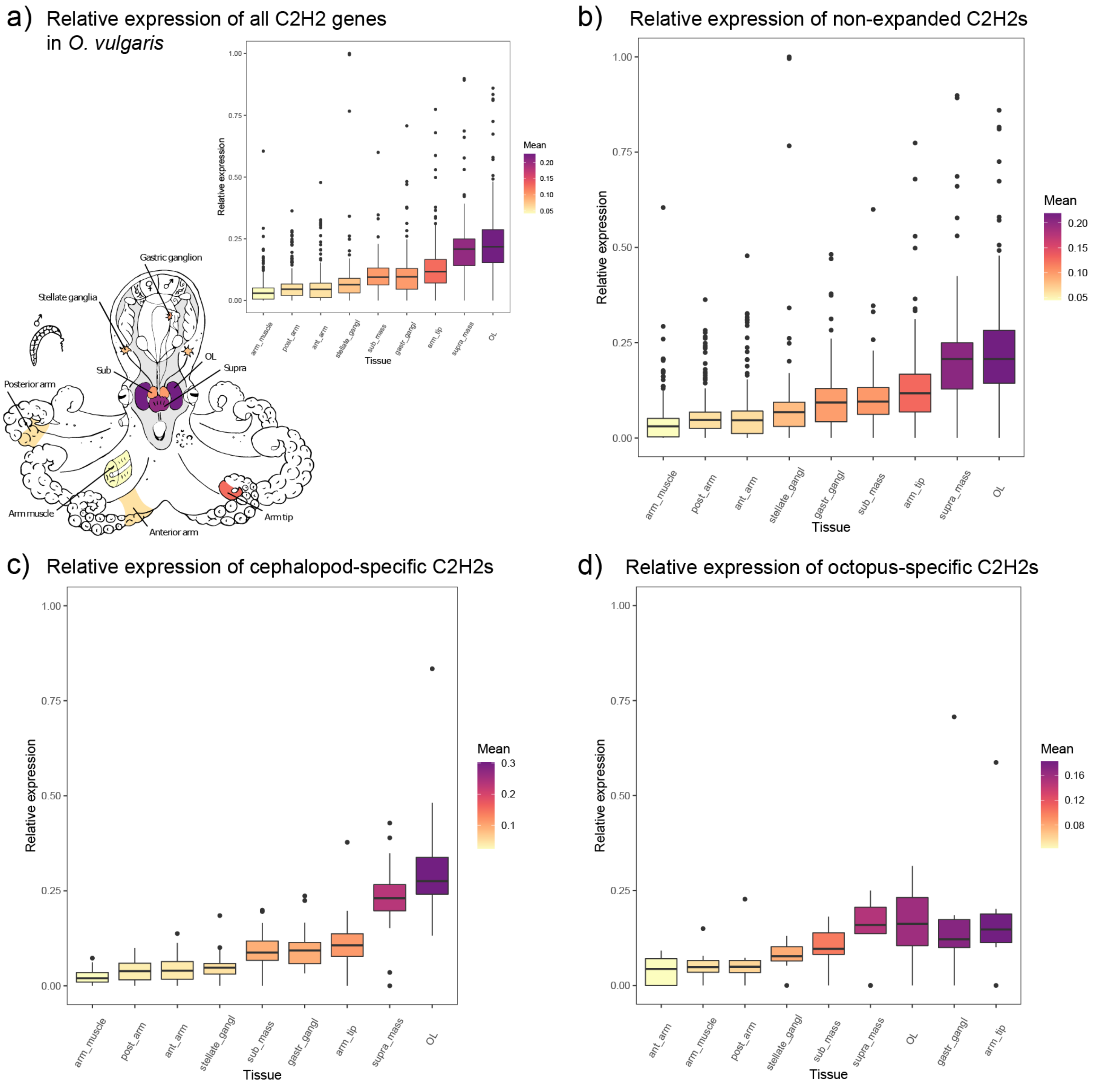

Figure S5. Relative expression of a) all C2H2 genes, b) non-expanded, c) cephalopod- and d) octopus- specific expanded C2H2s in *O. vulgaris*. Tissues in boxplots are sorted using their means, from lowest (left) to highest (right). a) The box colours were then used to colour the corresponding tissues in the animal sketch. Illustrations by Elena Ritschard.

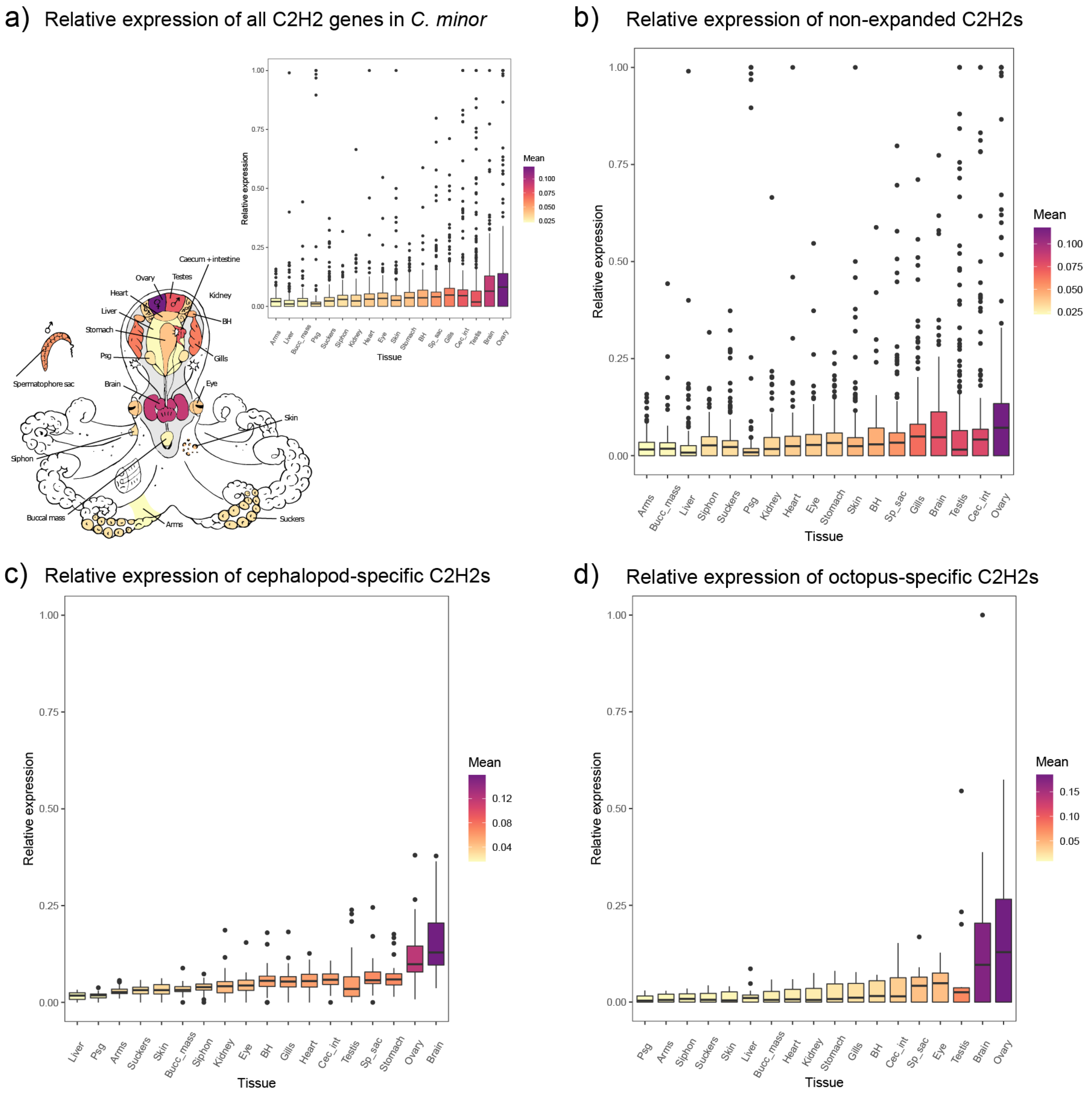

Figure S6. Relative expression of a) all C2H2 genes, b) non-expanded, c) cephalopod- and d) octopus- specific expanded C2H2s in *C. minor*. Tissues in boxplots are sorted using their means, from lowest (left) to highest (right). a) The box colours were then used to colour the corresponding tissues in the animal sketch. Illustrations by Elena Ritschard.

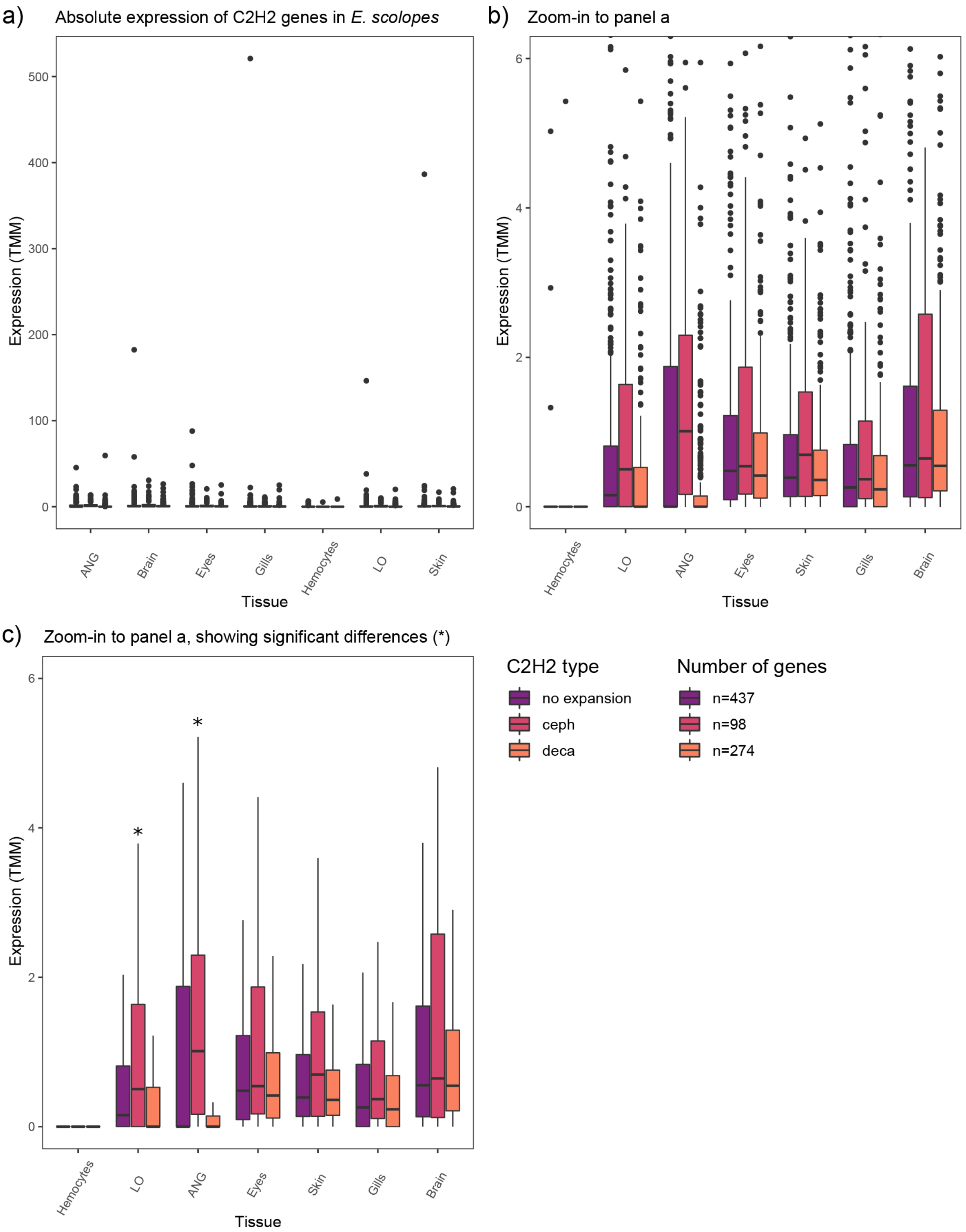

Figure S7. Absolute expression of *E. scolopes* non-expanded, cephalopod- and squid-expanded C2H2 genes in each tissue. Tissues are sorted using the group mean for each tissue, from lowest (left) to highest (right). a) Expression boxplot including all outliers. b) Zoom-in to panel *a* in the range 0 to 6 TMM expression values, c) Zoom-in to panel *a* in the range 0 to 6 TMM expression values but without outliers. Tissues with a significantly higher expression of

cephalopod-specific expanded C2H2 genes in comparison to both non- and squid-expanded genes were marked with an asterisk (*).

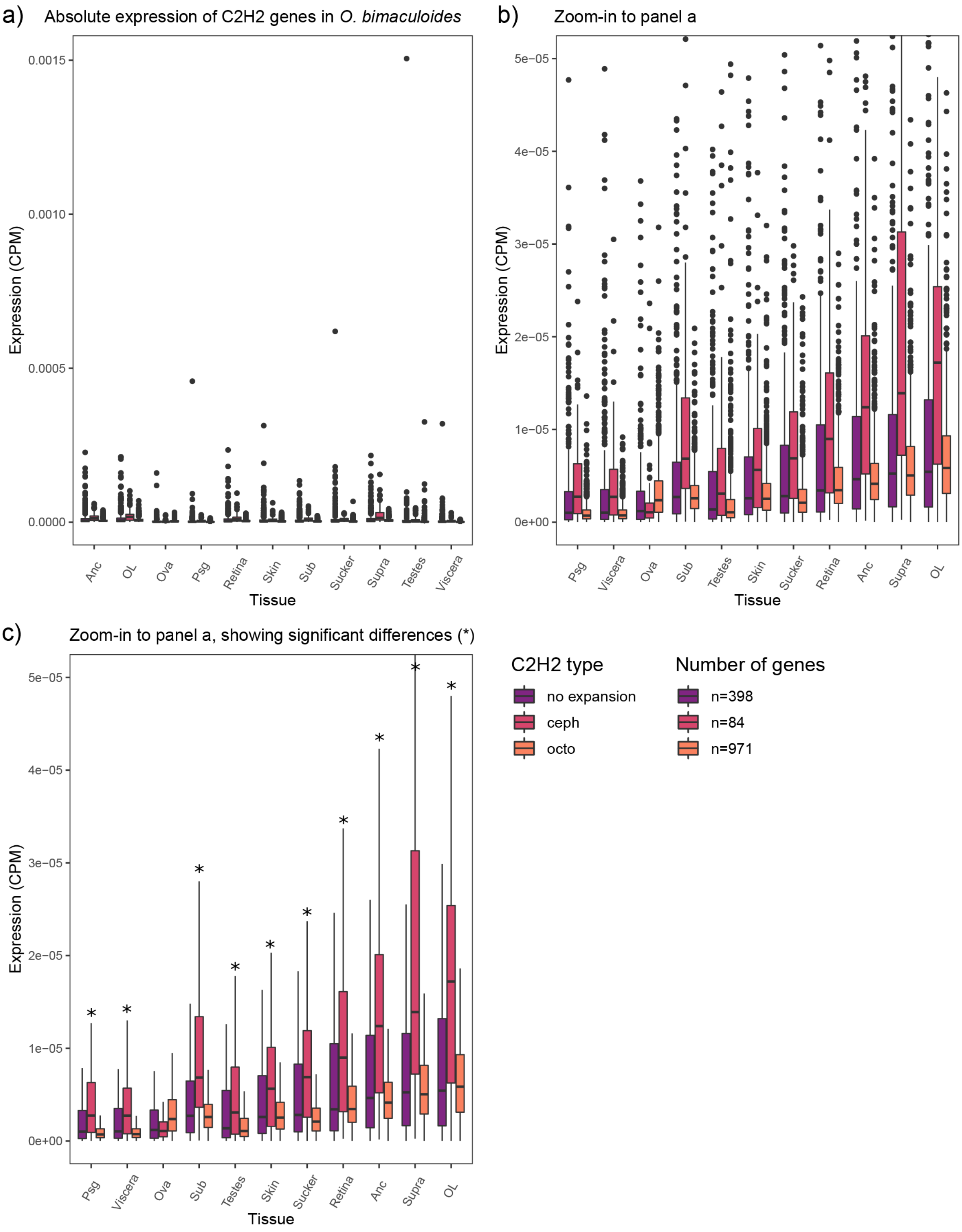

Figure S8. Absolute expression of *O. bimaculoides* non-expanded, cephalopod- and octopus-expanded C2H2 genes in each tissue. Tissues are sorted using the group mean for each tissue, from lowest (left) to highest (right). a) Expression boxplot including all outliers. b) Zoom-in to panel *a* in the range 0 to 5e-5 CPM expression values, c) Zoom-in to panel *a* in the range 0 to 5e-5 CPM expression values but without outliers. Tissues with a significantly

higher expression of cephalopod-specific expanded C2H2 genes in comparison to both non- and octopus-expanded genes were marked with an asterisk (*).

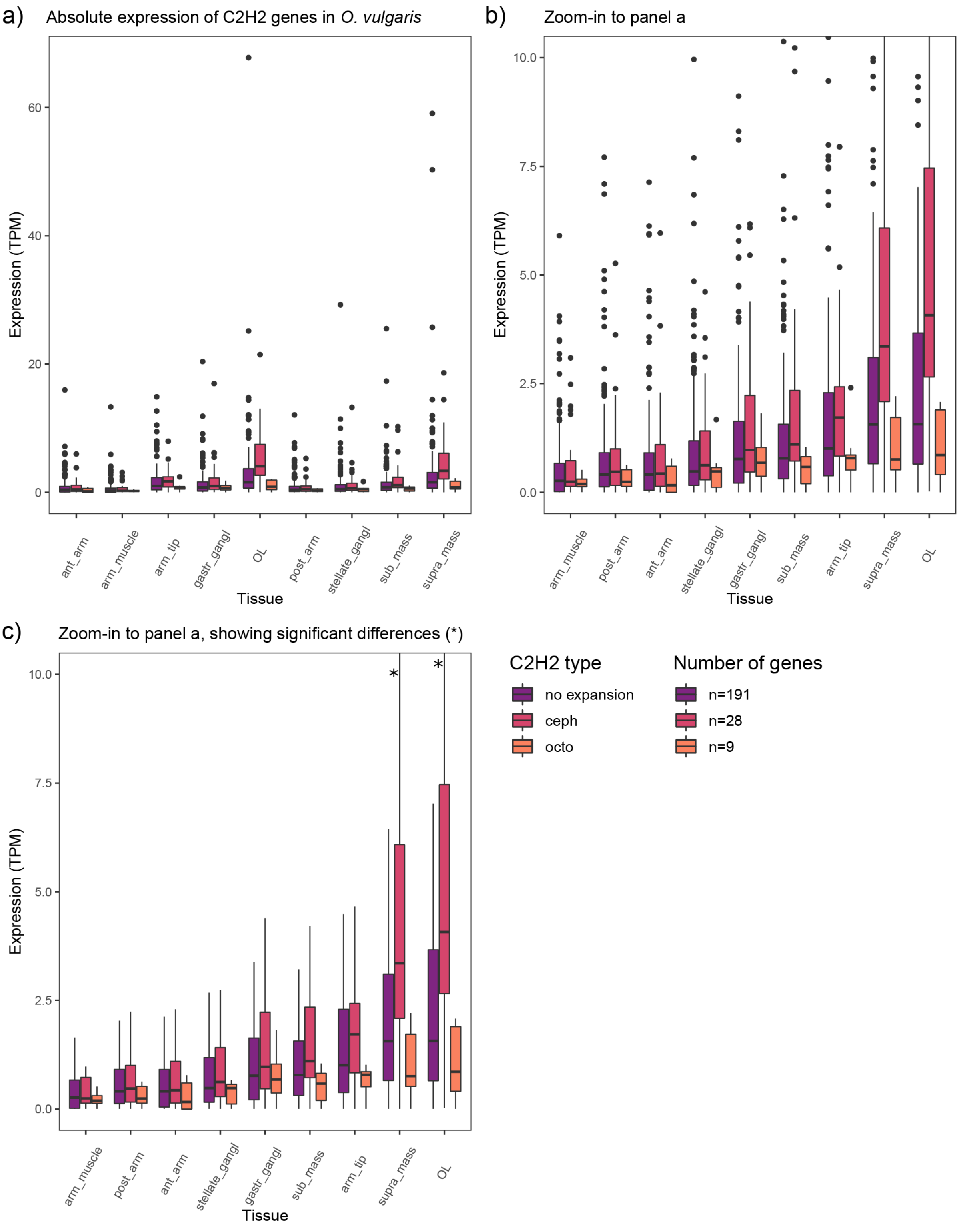

Figure S9. Absolute expression of *O. vulgaris* non-expanded, cephalopod- and octopus-expanded C2H2 genes in each tissue. Tissues are sorted using the group mean for each tissue, from lowest (left) to highest (right). a) Expression boxplot including all outliers. b) Zoom-in to panel *a* in the range 0 to 10 TPM expression values, c) Zoom-in to panel *a* in the range 0 to 10 TPM

expression values but without outliers. Tissues with a significantly higher expression of cephalopod-specific expanded C2H2 genes in comparison to both non- and octopus-expanded genes were marked with an asterisk (*).

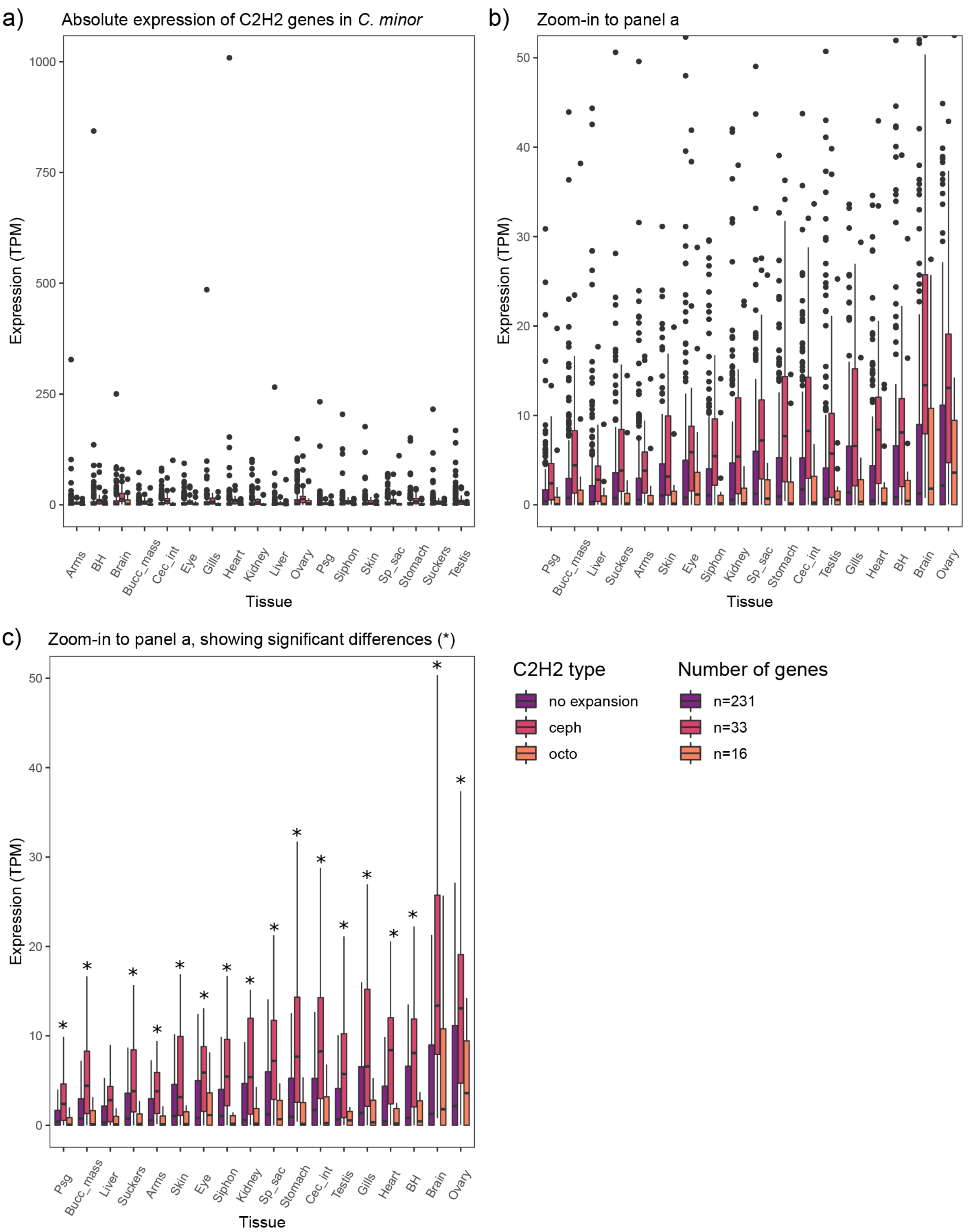

Figure S10. Absolute expression of *C. minor* non-expanded, cephalopod- and octopus-expanded C2H2 genes in each tissue. Tissues are sorted using the group mean for each tissue, from lowest (left) to highest (right). a) Expression boxplot including all outliers. b) Zoom-in to panel *a* in the range 0 to 50 TPM expression values, c) Zoom-in to panel *a* in the range 0 to 50 TPM expression values but without outliers. Tissues with a significantly higher expression of

cephalopod-specific expanded C2H2 genes in comparison to both non- and octopus-expanded genes were marked with an asterisk (*).

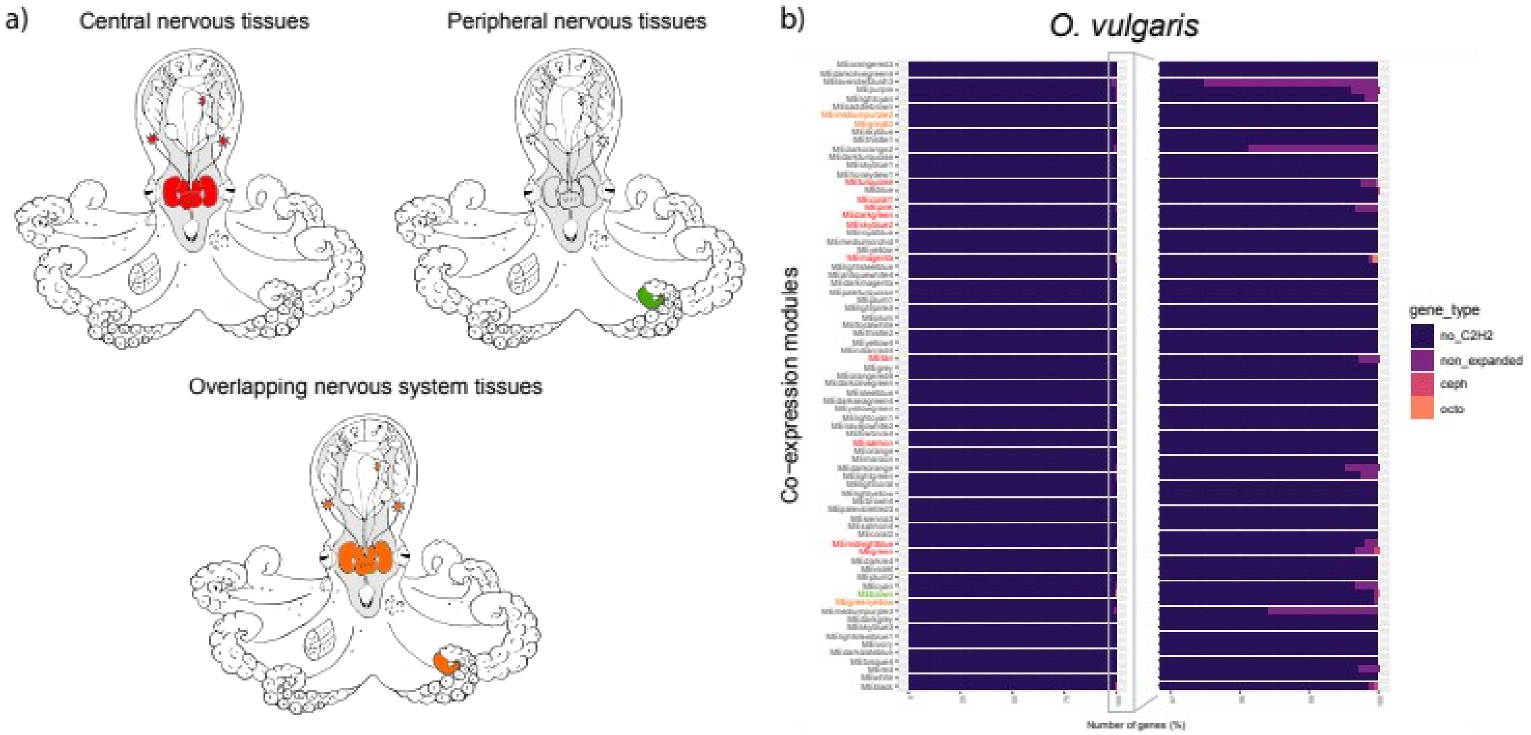

Figure S11. Distribution of C2H2 genes in the resulting WGCNA co-expression modules in *O. vulgaris*. Number of genes are shown as percentages (from 0% to 100% in left barplot and zoom-in to a shorter range in right barplot, for greater detail). Module names are color-coded based on their correlation to nervous tissues: red for central nervous tissues (brain, gastric ganglion and stellate ganglia), green for peripheral nervous tissues (arm tip) and orange for overlapping central and peripheral nervous tissues. Modules coloured black were not specifically correlated to nervous tissues.

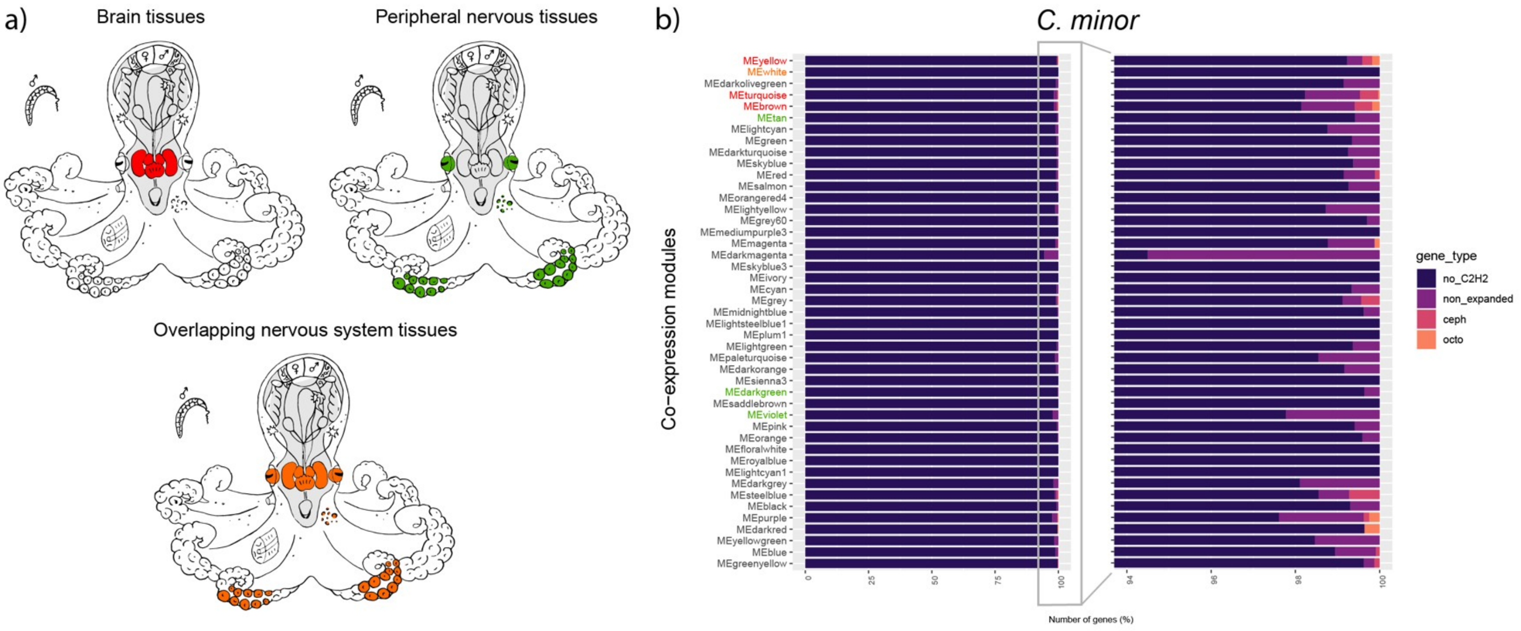

Figure S12. Distribution of C2H2 genes in the resulting WGCNA co-expression modules in *C. minor*. Number of genes are shown as percentages (from 0% to 100% in left barplot and zoom-in to a shorter range in right barplot, for greater detail). Module names are color-coded based on their correlation to nervous tissues: red for brain tissues, green for peripheral nervous tissues and orange for overlapping central and peripheral nervous tissues. Modules coloured black were not specifically correlated to nervous tissues.

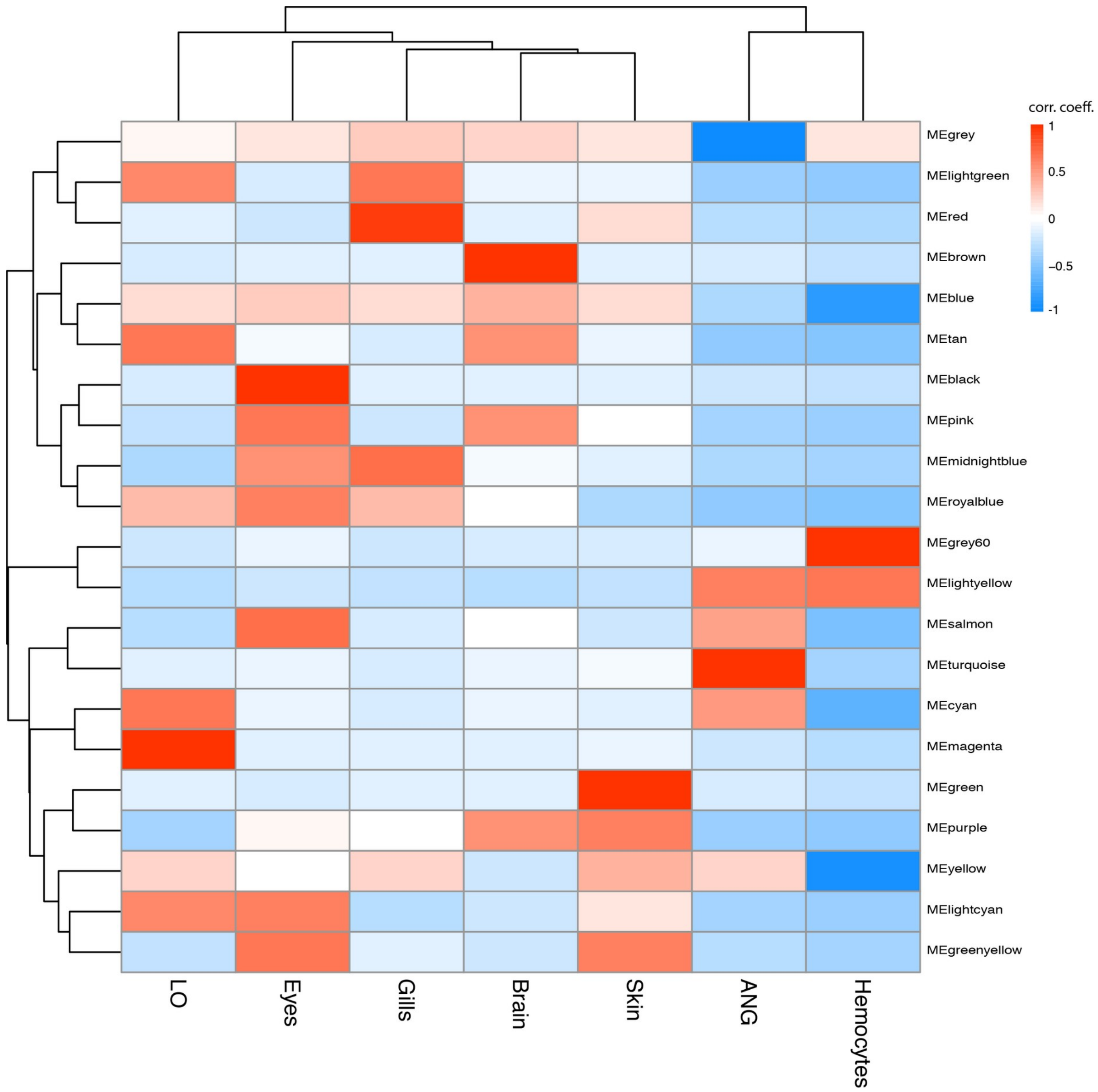

Figure S13. WGCNA module-trait relationship heatmap of *E. scolopes*. The heatmap visualizes the calculated correlation between eigengenes (y-axis), understood as the average expression profiles of the resulting modules, and each adult tissue (x-axis). Colours represent the correlation coefficient values, ranging from -1 (*i.e.,* negative correlation) to 1 (*i.e.,* positive correlation).

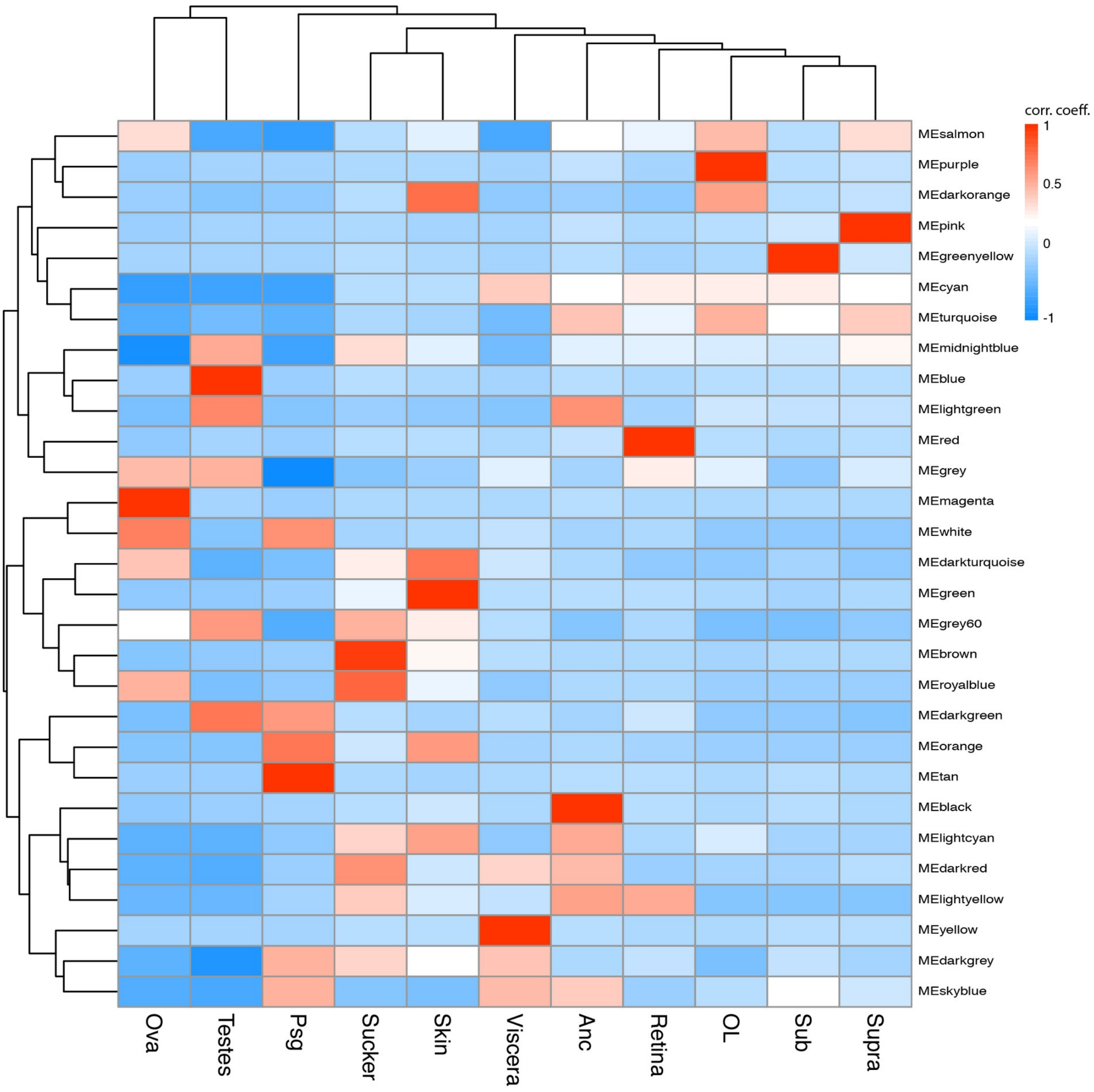

Figure S14. WGCNA module-trait relationship heatmap of *O. bimaculoides*. The heatmap visualizes the calculated correlation between eigengenes (y-axis), understood as the average expression profiles of the resulting modules, and each adult tissue (x-axis). Colours represent the correlation coefficient values, ranging from -1 (*i.e.,* negative correlation) to 1 (*i.e.,* positive correlation).

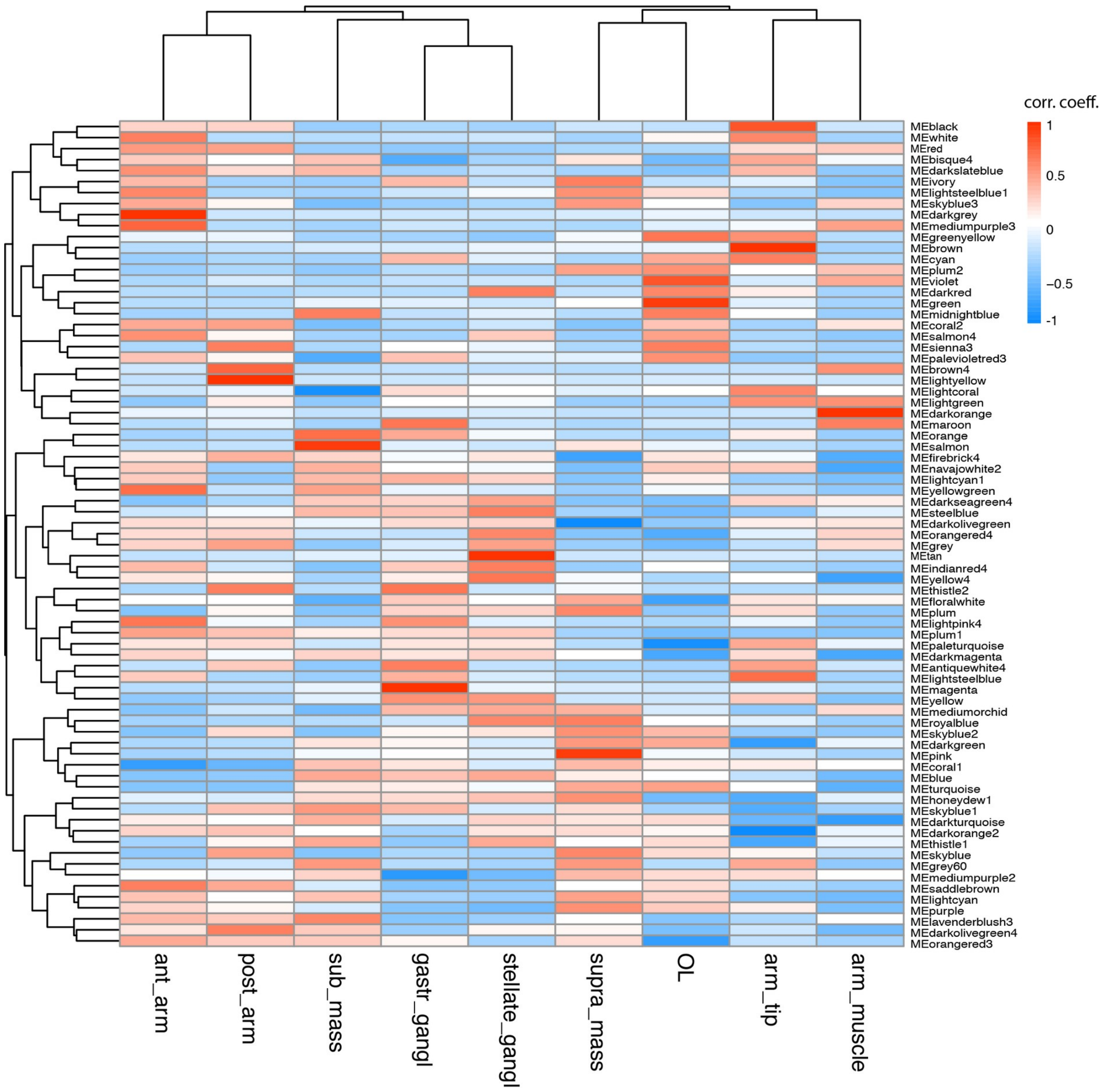

Figure S15. WGCNA module-trait relationship heatmap of *O. vulgaris*. The heatmap visualizes the calculated correlation between eigengenes (y-axis), understood as the average expression profiles of the resulting modules, and each adult tissue (x-axis). Colours represent the correlation coefficient values, ranging from -1 (*i.e.,* negative correlation) to 1 (*i.e.,* positive correlation).

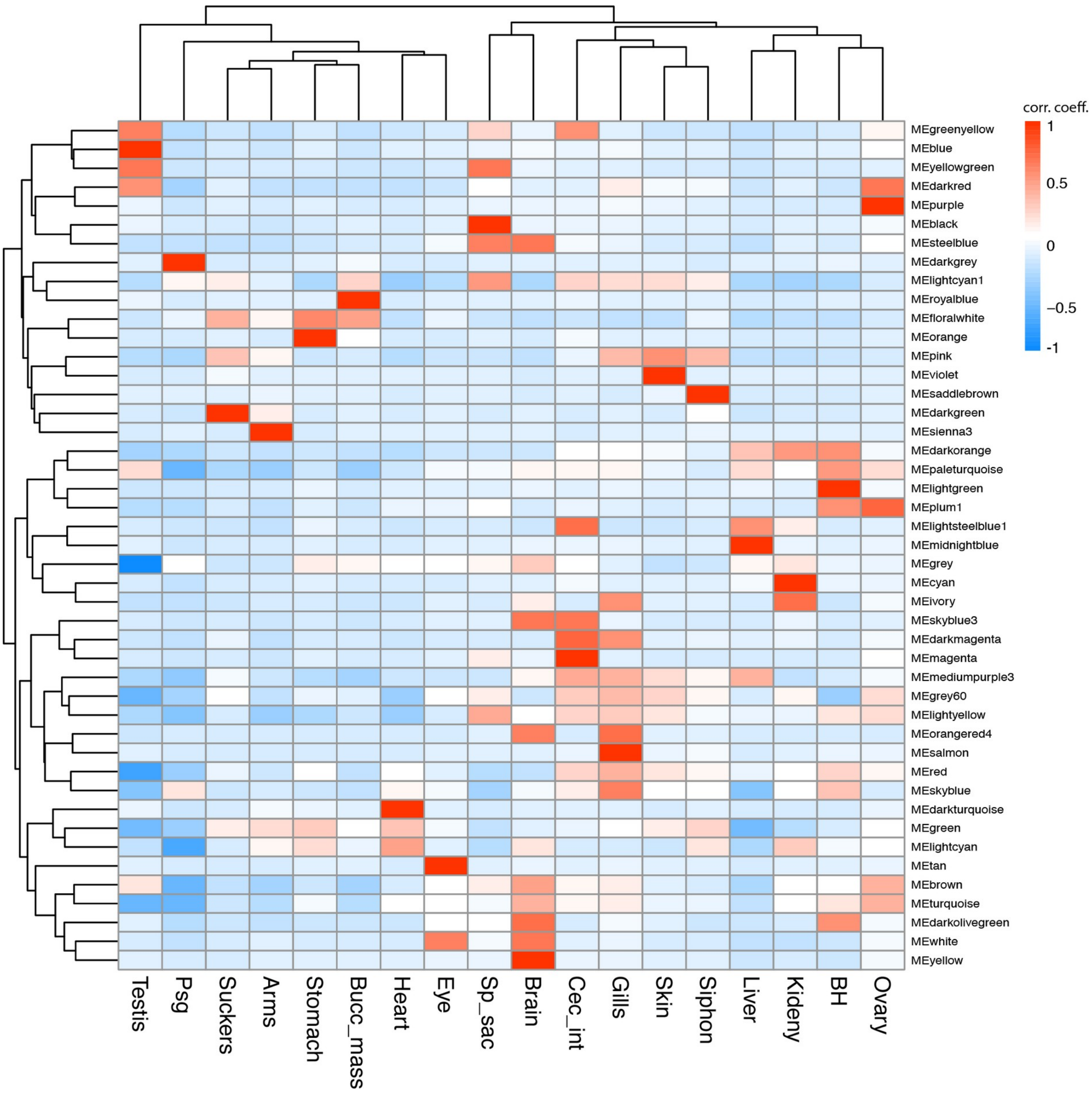

Figure S16. WGCNA module-trait relationship heatmap of *C. minor*. The heatmap visualizes the calculated correlation between eigengenes (y-axis), understood as the average expression profiles of the resulting modules, and each adult tissue (x-axis). Colours represent the correlation coefficient values, ranging from -1 (*i.e.,* negative correlation) to 1 (*i.e.,* positive correlation).

2. Tables

Table S1. Species used in the phylogenetic analysis and the corresponding source of their sequences. **Idiosepius paradoxus* dataset was taken from the NCBI database under the BioSample accession number SAMN00152410.

ABBREVIATION	SPECIES NAME	COMMON NAME	SOURCE	PROTEOME
**ANOGA**	*Anopheles gambiae*	African malaria mosquito	UniProt	Yes
**ARCDU**	*Architeuthis dux*	Giant squid	[S1]	Yes
**BRAFL**	*Branchiostoma floridae*	Florida lancelet	UniProt	Yes
**CAEEL**	*Caenorhabditis elegans*	Round worm	UniProt	Yes
**CALMI**	*Callistoctopus minor*	Long arm octopus	[S2]	Yes
**CAPTE**	*Capitella teleta*	Polychaete worm	UniProt	Yes
**CRAGI**	*Crassostrea gigas*	Pacific oyster	UniProt	No
**DORPE**	*Doryteuthis pealeii*	Longfin inshore squid	[S3]	Yes
**DROME**	*Drosophila melanogaster*	Fruit fly	UniProt	Yes
**EUPSC**	*Euprymna scolopes*	Hawaiian bobtail squid	[S4]	Yes
**HELRO**	*Helobdella robusta*	Californian leech	UniProt	Yes
**HUMAN**	*Homo sapiens*	Human	UniProt	Yes
**IDIPA**	*Idiosepius paradoxus*	Northern pygmy squid	NCBI*	Yes
**LOTGI**	*Lottia gigantea*	Giant owl limpet	UniProt	Yes
**MIZYE**	*Mizuhopecten yessoensis*	Japanese scallop	UniProt	Yes
**OCTBI**	*Octopus bimaculoides*	California two-spot octopus	[S5]	Yes
**OCTVU**	*Octopus vulgaris*	Common octopus	[S6]	Yes
**SACKO**	*Saccoglossus kowalevskii*	Acorn worm	UniProt	No
**SCHMA**	*Schistosoma mansoni*	Blood fluke	UniProt	Yes
**STRPU**	*Strongylocentrotus purpuratus*	Purple sea urchin	UniProt	Yes

Table S2. Tissues from the different cephalopod species available as expression data and used in this study. "X" means data available. Similar tissues were grouped together and sorted starting with the most relevant ones for this study (*i.e.,* brain, eye, ganglia, skin and arm tissues). Species abbreviations are as follows: CALMI: *C. minor,* EUPSC: *E. scolopes,* OCTBI: *O. bimaculoides*, OCTVU: *O. vulgaris*. Tissue abbreviations are as followed: ANC: axial nerve cord, ANG: accessory nidamental gland, ant_arm: anterior arm, post_arm: posterior arm, BH: branchial heart, OL: optic lobe, sub: subesophageal brain, supra: supraesophageal brain, Bucc_mass: buccal mass, Cec_int: caecum intestine, Gastr_gangl: gastric ganglion, heart: sytemic heart, LO: light organ, Psg: posterior salivary gland, Sp_sac: spermatophore sac, Stellate_gangl: stellate ganglion and viscera: heart, kidney, and hepatopancreas.

TISSUES:	CALMI	EUPSC	OCTBI	OCTVU
**BRAIN**	X	X		
**OL**			X	X
**SUB**			X	X
**SUPRA**			X	X
**EYES**	X	X		
**RETINA**			X	
**GASTR_GANGL**				X
**STELLATE_GANGL**				X
**SKIN**	X	X	X	
**ARM**	X			
**ANT_ARM**				X
**POST_ARM**				X
**ARM_TIP**				X
**ARM_MUSCLE**				X
**ANC**			X	
**SUCKER**	X		X	
**ANG**		X		
**BH**	X			
**BUCC_MASS**	X			
**CEC_INT**	X			
**GILLS**	X	X		
**HEART**	X			
**HAEMOCYTES**		X		
**KIDNEY**	X			
**LIVER**	X			
**LO**		X		
**OVARY**	X		X	
**PSG**	X		X	

**SIPHON**	X	
**SP_SAC**	X	
**STOMACH**	X	
**TESTIS**	X	X
**VISCERA**		X

Table S3. Number of sequences in the resulting expansion clusters. Cluster ID recalls whether the expansion was characterized as cephalopod-, octopus- or squid-specific. Column named “S-H support” stands for the support value of the cluster in the phylogenetic tree using the Shimodaira-Hasegawa test as described in the Methods. Species abbreviations are as follows: ARCDU: *Architeuthis dux*, DORPE: *Doryteuthis pealeii*, EUPSC: *Euprymna scolopes*, IDIPA: *Idiosepius paradoxus*, CALMI: *Callistoctopus minor*, OCTBI: *Octopus bimaculoides*, OCTVU: *Octopus vulgaris.*

CLUSTER ID	S-H SUPPORT	TOTAL NR. OF SEQUENCES	SQUID	OCTOPUS	ARCDU	DORPE	EUPSC	IDIPA	CALMI	OCTBI	OCTVU
**CEPH 1**	0,236	163	106	57	31	37	38	0	11	35	11
**CEPH 2**	0,835	132	89	43	26	28	35	0	6	27	10
**CEPH 3**	0,809	76	46	30	15	16	15	0	11	15	4
**CEPH 4**	0,989	39	24	15	7	7	10	0	5	7	3
**OCTO 1**	0,465	495	1	494	1	0	0	0	0	494	0
**OCTO 2**	0,787	235	1	234	0	1	0	0	9	223	2
**OCTO 3**	0,851	167	3	164	0	1	2	0	1	157	6
**OCTO 4**	0,814	104	0	104	0	0	0	0	6	97	1
**SQUID 1**	0,767	984	984	0	4	882	96	2	0	0	0
**SQUID 2**	0,778	499	499	0	0	415	82	2	0	0	0
**SQUID 3**	0,887	161	161	0	0	144	17	0	0	0	0
**SQUID 4**	0,771	107	107	0	0	106	1	0	0	0	0
**SQUID 5**	0,845	47	47	0	0	47	0	0	0	0	0
**SQUID 6**	0,888	46	44	2	0	36	8	0	0	1	1
**SQUID 7**	0,864	42	42	0	0	1	41	0	0	0	0
**SQUID 8**	0	36	36	0	0	33	3	0	0	0	0
**SQUID 9**	0,832	28	28	0	0	5	23	0	0	0	0
**SQUID 10**	0,962	25	25	0	0	22	3	0	0	0	0
**SQUID 11**	0,794	20	20	0	0	20	0	0	0	0	0

Table S4. Summary of GO term analysis results. Expression modules used in the GO term enrichment analysis (1st column), their correlated tissues (2nd column), the total number of genes per module (3rd column), the number of lineage-specific (squid- or octopus-specific), cephalopod-specific and non-expanded C2H2 genes (4th, 5th and 6th column). The last three columns show whether the C2H2 GO:0003676 and the GPCR GO:0007186 were enriched ("y" for yes, "n" for no), and how often the GPCR GO term was found in the respective module. Tissue abbreviations are as follows: ANC: axial nerve cord, OL: optic lobe, supra: supraesophageal brain, Gastr_gangl: gastric ganglion, LO: light organ, Sp_sac: spermatophore sac. Species abbreviations associated with module names are as follows: EUPSC for *E. scolopes,* CALMI for *C. minor,* OCTBI for *O. bimaculoides* and OCTVU for *O. vulgaris.*

Modules	Tissue	Size	Squid /Octo	Ceph	Non-exp	C2H2 enriched	GPCR enriched	# GPCR GO term
**EUPSCpurple**	Skin, Brain	596	28	2	24	y	y	17
**EUPSCpink**	Eyes, Brain	705	33	0	40	y	n	0
**EUPSCtan**	LO, Brain	325	10	2	10	n	y	12
**CALMIsteelblue**	Brain, Sp_sac	138	0	1	1	n	y	7
**CALMIbrown**	Brain, Ovary	2205	4	9	28	y	n	0
**CALMIturquoise**	Brain, Ovary	2986	1	13	39	y	n	0
**OCTBIsalmon**	OL, Supra, Ovary	507	151	1	25	y	n	0
**OCTBIturquoise**	OL, Supra, Anc	11164	646	64	224	y	n	0
**OCTBIgrey60**	Sucker, Testis	59	3	0	0	n	n	0
**OCTVUmagenta**	Gastr_gangl	1288	1	0	1	n	y	6
**OCTVUgreen**	OL	5013	0	3	15	n	y	19

**OCTVUturquoise**	OL, Supra	57662	7	23	121	y	y	120

References

S1. R. R. Da Fonseca, A. Couto, A. M. Machado, B. Brejova, C. B. Albertin, F. Silva, P. Gardner, T. Baril, A. Hayward, A. Campos, Â. M. Ribeiro, I. Barrio-Hernandez, H. J. Hoving, R. Tafur-Jimenez, C. Chu, B. Frazão, B. Petersen, F. Peñaloza, F. Musacchia, G. C. Alexander, H. Osório, I. Winkelmann, O. Simakov, S. Rasmussen, M. Z. Rahman, D. Pisani, J. Vinther, E. Jarvis, G. Zhang, J. M. Strugnell, L. F. C. Castro, O. Fedrigo, M. Patricio, Q. Li, S. Rocha, A. Antunes, Y. Wu, B. Ma, R. Sanges, T. Vinar, B. Blagoev, T. Sicheritz-Ponten, R. Nielsen, M. T. P. Gilbert, A draft genome sequence of the elusive giant squid, Architeuthis dux. Gigascience. 9, 1–12 (2020).

S2. B. M. Kim, S. Kang, D. H. Ahn, S. H. Jung, H. Rhee, J. S. Yoo, J. E. Lee, S. Lee, Y. H. Han, K. Bin Ryu, S. J. Cho, H. Park, H. S. An, The genome of common long-arm octopus Octopus minor. Gigascience. 7, 1–7 (2018).

S3. C.B. Albertin, S. Medina-Ruiz, T. Mitros, H. Schmidbaur, G. Sanchez, Z. Y. Wang, J. Grimwood, J. J. C. Rosenthal, C. W. Ragsdale, O. Simakov, D. S. Rokshar. Genome and transcriptome mechanisms driving cephalopod evolution. Nat Commun 13, 2427 (2022). doi:10.1038/s41467-022-29748-w

S4. M. Belcaid, G. Casaburi, S. J. McAnulty, H. Schmidbaur, A. M. Suria, S. Moriano-Gutierrez, M. Sabrina Pankey, T. H. Oakley, N. Kremer, E. J. Koch, A. J. Collins, H. Nguyen, S. Lek, I. Goncharenko-Foster, P. Minx, E. Sodergren, G. Weinstock, D. S. Rokhsar, M. McFall-Ngai, O. Simakov, J. S. Foster, S. V. Nyholm, Symbiotic organs shaped by distinct modes of genome evolution in cephalopods. Proc. Natl. Acad. Sci. U. S. A. 116, 3030–3035 (2019).

S5. C. B. Albertin, O. Simakov, T. Mitros, Z. Y. Wang, J. R. Pungor, E. Edsinger-Gonzales, S. Brenner, C. W. Ragsdale, D. S. Rokhsar, The octopus genome and the evolution of cephalopod neural and morphological novelties. Nature. 524, 220–224 (2015), doi:10.1038/nature14668

S6. I. Zarrella, K. Herten, G. E. Maes, S. Tai, M. Yang, E. Seuntjens, E. A. Ritschard, M. Zach, R. Styfhals, R. Sanges, O. Simakov, G. Ponte, G. Fiorito, The survey and reference assisted assembly of the Octopus vulgaris genome. Sci. Data. 6, 1–8 (2019).
